# Efficient Water Splitting Cascade Photoanodes with Ligand‐Engineered MnO Cocatalysts

**DOI:** 10.1002/advs.201800727

**Published:** 2018-08-06

**Authors:** Mi Gyoung Lee, Kyoungsuk Jin, Ki Chang Kwon, Woonbae Sohn, Hoonkee Park, Kyoung Soon Choi, Yoo Kyung Go, Hongmin Seo, Jung Sug Hong, Ki Tae Nam, Ho Won Jang

**Affiliations:** ^1^ Department of Materials Science and Engineering Research Institute of Advanced Materials Seoul National University Seoul 08826 Republic of Korea; ^2^ Advanced Nano Surface Research Group Korea Basic Science Institute Daejeon 34133 Republic of Korea

**Keywords:** band structure, ligand engineering, MnO, oxygen evolution catalysts, water splitting

## Abstract

The band edge positions of semiconductors determine functionality in solar water splitting. While ligand exchange is known to enable modification of the band structure, its crucial role in water splitting efficiency is not yet fully understood. Here, ligand‐engineered manganese oxide cocatalyst nanoparticles (MnO NPs) on bismuth vanadate (BiVO_4_) anodes are first demonstrated, and a remarkably enhanced photocurrent density of 6.25 mA cm^−2^ is achieved. It is close to 85% of the theoretical photocurrent density (≈7.5 mA cm^−2^) of BiVO_4_. Improved photoactivity is closely related to the substantial shifts in band edge energies that originate from both the induced dipole at the ligand/MnO interface and the intrinsic dipole of the ligand. Combined spectroscopic analysis and electrochemical study reveal the clear relationship between the surface modification and the band edge positions for water oxidation. The proposed concept has considerable potential to explore new, efficient solar water splitting systems.

## 1 Introduction

The growing social demand for energy poses many challenges, such as increasing energy efficiency, developing new energy supplies, and protecting the environment. Hydrogen is considered as an ideal energy carrier to meet these challenges since it is clean, renewable, carbon‐free, and has a high energy density. Photo‐electrochemical (PEC) hydrogen production by water splitting stockpiles solar energy into chemical bonds and is thus considered as a key technology of the future, attracting widespread attention.^1^, ^2^, ^3^, ^4^, ^5^, ^6^, ^7^, ^8^

Bismuth vanadate (BiVO_4_), which is an n‐type semiconductor, has rapidly emerged as a most promising photoanode in PEC cells since it absorbs a substantial portion of the visible spectrum (bandgap energy, ≈2.4 eV) and has a favorable valence band position for water oxidation.^4^, ^5^, ^7^, ^8^, ^9^, ^10^, ^11^, ^12^ However, the solar hydrogen conversion efficiency achieved with BiVO_4_ to date has been lower than theoretically expected, because BiVO_4_ suffers from poor electron–hole separation yield and tardy reaction kinetics on the surface.^4^, ^8^, ^10^, ^12^ In this regard, elemental doping,^11^ nanostructuring,^4^ heterojunctions,^4^, ^12^, ^13^, ^14^, ^15^, ^16^, ^17^, ^18^ and crystal facet^19^, ^20^ have been attempted to improve the photoactivity of BiVO_4_ anodes. In our previous work, we introduced nanostructured heterojunction anode to overcome limitation of electron/hole recombination.^4^ We achieved the photocurrent density of 4.55 mA cm^−2^ at 1.23 V versus reversible hydrogen electrode (RHE). Despite the substantial improvement of photoactivity of BiVO_4_‐based anodes, it is still lower than theoretical photocurrent density of 7.5 mA cm^−2^. Sluggish oxygen evolving reaction (OER) kinetics on anode surface, hole accumulation at the photoanode/electrolyte interface (photocorrosion), and trap states have been considered as unsolved issues for performance of BiVO_4_‐based photoanodes. Therefore, another approach is needed to overcome these drawbacks and reach a practical solution of this issue.

Surface modification with oxygen evolution catalysts (OECs) would be an innovative approach to solve the above issues. Integration of electrocatalyst as OECs for OER onto the surface of the photoelectrode often improves performance of the device. The common function of the catalyst on a semiconductor surface can be categorized as follows: 1) to catalyze the bond‐making and bond‐breaking reactions; 2) to passivate the recombination sites; 3) to tune the band structure energetics; and 4) to protect the surface from corrosion.^9^, ^11^, ^12^, ^21^, ^22^, ^23^, ^24^, ^25^, ^26^, ^27^, ^28^ Although IrO_2_ and RuO_2_ have been commonly utilized as OECs, the moderate activity and scarce nature of precious metals limit the widespread use of these catalysts for PEC water oxidation. Instead, transition metal–based OECs such as Co–Pi, FeOOH, and NiOOH have been recently reported due to their low cost, proper band positions with BiVO_4_, and good activity under benign conditions (neutral pH range, room temperature).^29^, ^30^

The use of Mn‐based catalysts for efficient water oxidation has been widely studied, due to the presence of biological water oxidizing complex in photosystem II, which comprises four manganese and one calcium atoms. Moreover, high cost‐effectiveness (Mn is the 10th most abundant element in the earth's crust), low toxicity, and robust stability in neutral condition are other attractive properties of a manganese‐based catalyst as OECs.^31^, ^32^, ^33^, ^34^, ^35^, ^36^ In addition, the multioxidation states of manganese‐based catalyst would facilitate the local hole transport around Mn centers via a low barrier of O—Mn—O pathway, which is likely to boost the water oxidation activity.^33^ Specifically, under neutral condition, manganese oxide nanoparticles exhibited superior performance to previously reported transition metal–based catalysts.^32^, ^33^ Several researches show that Mn‐based oxides have superior catalytic activity at neutral pH and are highly active in water oxidation compared to low activity oxides, as shown in Table S1 (Supporting Information).^31^, ^32^, ^37^, ^38^, ^39^, ^40^, ^41^, ^42^, ^43^, ^44^, ^45^, ^46^, ^47^, ^48^, ^49^ As compared to other OECs, Mn‐based oxides showed good catalytic properties (Tafel slope and overpotential) in the neutral pH condition for water oxidation. From these data, we can know that Mn‐based catalyst can be a good candidate for solar water oxidation. In this work, as an effort to enhance photoactivities, we utilized manganese oxide nanoparticles (MnO NPs) on BiVO_4_‐based photoanodes as a cocatalyst.

While the surface of BiVO_4_‐based anodes is poorly catalytic for water oxidation, several studies such as control of the morphology and composition of BiVO_4_ itself to solve poorly catalytic reaction have failed to directly result in photocurrent enhancement.^10^, ^13^ So far, studies on OECs/BiVO_4_ commonly reported that the presence of OECs enhanced photoactivities due to reduction of the surface recombination by efficient hole withdrawal from the BiVO_4_ layer and efficient hole consumption for water oxidation. However, the overall performance of OEC/BiVO_4_ is significantly governed by the OEC/BiVO_4_ interface, thus better understanding on junction of photoanode/OEC is necessary for further optimization of BiVO_4_‐based photoelectrodes. Therefore, we believe it is necessary to focus on the design of efficient OECs on the BiVO_4_ surface with proper junction.

The catalyst–substrate interaction can effectively modify the binding energy of the reaction intermediated through either electronic perturbation of the active sites on the surface or direct participation in the reaction.^28^ Previously, it has been documented that surface modification as ligand exchange could enable to tune the Helmholtz double layer through the adsorption of charged species on the surface of the several materials such as quantum dot (QD) for solar cell, and consequently shift band edge energies.^50^, ^51^, ^52^, ^53^, ^54^, ^55^, ^56^, ^57^, ^58^ All of these studies demonstrated that modifying the ligand/QD interface produces quite distinct chemical systems, and some even suggested a link between QD band edge energy shifts and ligand dipole moment; however, a clear and quantitative relationship between ligand exchange and band edge shifts has never been reported. Changing the identity of the chemical binding group and dipole moment of the ligand also modulate the strength of the MnO–ligand surface dipole, shifting the vacuum energy and, in turn valence band maximum (VBM) and conduction band minimum (CBM) of MnO.^50^, ^52^, ^54^, ^55^ Induced dipole at the ligand/MnO interface and the intrinsic dipole of the ligand can move band edge energies toward the desirable direction for water oxidation.^51^, ^52^, ^54^ In other words, it is possible to promote separation of charge carriers without recombination at the interface when the band structure permits the efficient transport of photoexcited charge carriers, since ligand engineering would influence band edge positions. In addition, the type of appropriate ligands has a crucial role in increasing the charge transfer due to passivated electronic trap sites on the MnO surface. Even though, as described above, ligand exchange has a remarkable impact on energy level modification for cascade photoelectrode structures, no in‐depth study has yet been attempted to bring out the underlined mechanism for improving the PEC properties of BiVO_4_‐based photoanodes by introducing the ligand‐engineered MnO NPs. We use a combination of experiment and analysis to directly establish the mechanism of surface chemistry–induced band shifting for desirable solar water splitting.

Herein, we report the substantially enhanced photoactivities of BiVO_4_‐based anodes, decorated with the ligand‐engineered MnO NPs. The optimum MnO NPs decorated on BiVO_4_‐based anode achieved a photocurrent density of 6.25 mA cm^−2^ at 1.23 V versus RHE, which is more than 80% of theoretical photocurrent density of BiVO_4_ anodes. We stress that type of ligand molecules of MnO NPs dramatically affects the charge transfer efficiency of BiVO_4_‐based anodes by modifying the band edge positions of MnO NPs, and thus improves water oxidation property. From the combined spectroscopic studies, we proved the effect of diverse surface ligands on the band position and consequent photoactivities of BiVO_4_‐based anodes. We highlight that these findings are the innovative concepts of control over the PEC properties of the BiVO_4_‐based anodes through introduction of ligand‐engineered OECs in the presence of hole scavenger.

## 2 Results and Discussion

Sub‐10 nm sized MnO NPs were prepared by previously reported hot‐injection methods.^32^, ^33^, ^35^ During the synthesis procedure, MnO NPs are covered with fatty acid surfactants, myristic acids, which are used to maintain uniform sized particles. Since these ligands are nonconductive, post surface treatment is required. In order to remove the nonconducting ligands, we could either directly burn the ligands by annealing at higher temperature or exchange them with other conducting ligands. Since it is likely to occur phase change during annealing process, in this work, we chose to utilize the ligand exchange methodology to replace undesirable ligands on the MnO surface with conducting one.

**Figure 1:**
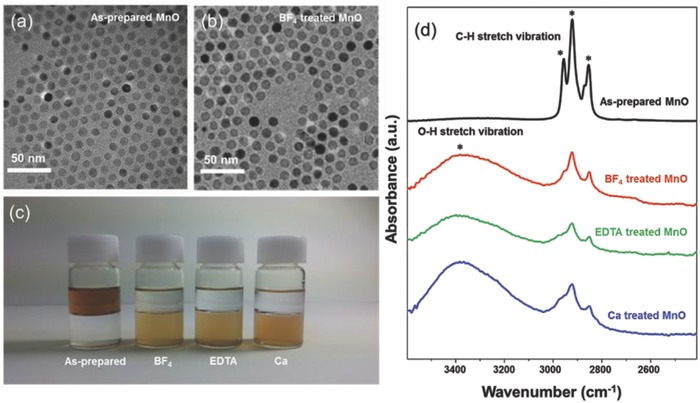
a,b) TEM image of 10 nm sized as‐prepared and BF_4_‐treated MnO crystals. c) Photographic images of the as‐prepared MnO, BF_4_‐treated, EDTA‐treated, and Ca–EDTA‐treated MnO NP solution. d) FT‐IR spectra of the as‐prepared, BF_4_‐treated, EDTA‐treated, and Ca–EDTA‐treated MnO.

Various conducting ligands such as tetrafluoroborate (BF_4_^−^), ethylenediaminetetraacetic acid (EDTA), Ca‐inserted EDTA, and ammonium (NH_4_^+^) are employed to anchor the MnO NPs. Capping ligand molecules on nanoparticle surface have played important role in functionalizing nanoparticles. However, in order to make monodisperse nanoparticles by thermal decomposition method, the insulating long‐chain organic ligands have been used. These ligands inevitably have disadvantage in interparticle charge transport between nanocrystals, which could be critical limitation on electrochemical/photo‐electrochemical reaction. In this regard, we tried to replace nonconducting ligand molecules with short inorganic functional ligands. Compared to myristic acid‐decorated MnO nanoparticles, EDTA‐ or BF_4_‐functionalized ones have enhanced electric conductivity and charge transport capability. The successful ligand exchange of MnO was investigated using various methods. Transmission electron microscopy (TEM) analysis showed the as‐prepared and after ligand exchange process of MnO NPs, as shown in **Figure** **1**a,b and Figure S1 (Supporting Information), respectively. No appreciable change was observed in the shape, uniformity, and dispersity of MnO NPs after ligand exchange. Moreover, it is confirmed that while initially dispersed in nonpolar solvent, hexane, MnO NPs are clearly dispersed in polar solvent such as deionized water (DI), methanol, and ethanol after surface treatment. Figure 1c shows photographs of the as‐prepared solution of MnO NPs, BF_4_‐treated, EDTA‐treated, and Ca–EDTA‐treated solution of MnO NPs. The as‐prepared solution of MnO NPs showed a dark brown color. The color of the MnO solution changed to a light brown color after ligand exchange. Depending on the type of the ligand of MnO, the color of the solution slightly changed. Fourier transform infrared spectroscopy (FT‐IR) analysis was conducted to monitor the water and ligand molecules on the NP surface, as shown in Figure 1d. The results indicated that most of functional myristic acid molecules on MnO NPs are removed. The FT‐IR spectra of the MnO NPs revealed that the intensity of the characteristic C—H stretching vibrations at 2850–2950 cm^−1^ corresponding to myristic acid molecules significantly decreased after the ligand exchange process. The broad spectral features around 3400 cm^−1^ appearing after surface treatment are assigned to the solvated water molecules, consistent with the hydrophilic nature of the MnO NPs. Also, the composition and the surface electronic states of the MnO NPs with different ligands were explored by X‐ray photoelectron spectroscopy (XPS) analysis, as shown in Figure S2 (Supporting Information). The two peaks at 641.2 and 652.8 eV for Mn 2p_3/2_ and Mn 2p_1/2_, respectively, are characteristic features of MnO NPs; this is also confirmed by the X‐ray diffraction (XRD) analysis, as displayed in Figure S3 (Supporting Information). After the ligand exchange of MnO such as Ca, Ca–EDTA, and BF_4_, the peaks are slightly shifted to the lower binding energies, indicating the slight reduction of manganese during the ligand exchange process.

**Figure 2:**
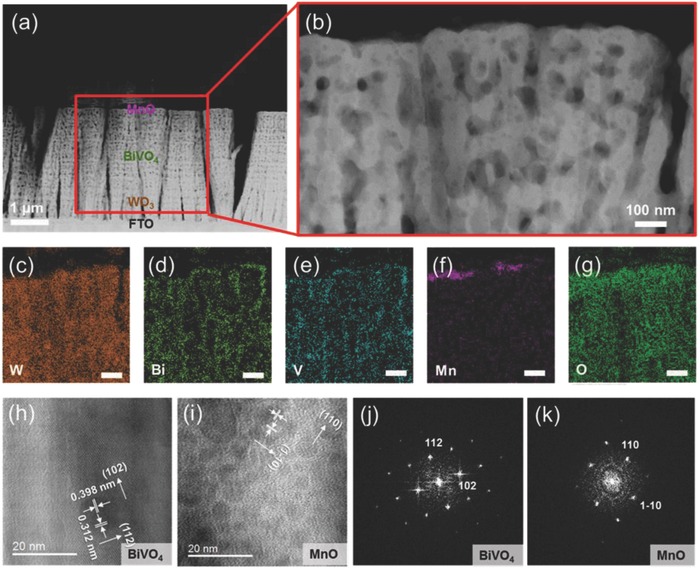
a) The corresponding TEM images of BF_4_‐treated MnO/BiVO_4_/WO_3_ nanorods. b) Expanded image of BF_4_‐treated MnO/BiVO_4_/WO_3_ nanorods. c–g) EDS images of W, Bi, V, Mn, and O, respectively. h) (112) and (102) of BiVO_4_. i) Small square by HR‐TEM shows crystalline planes of (110) and (1−10) of BF_4_‐treated MnO. j,k) Fast Fourier transform (FFT) pattern of BiVO_4_ and BF_4_‐treated MnO (* (c)–(g) scale bar is 100 nm).

MnO NP/BiVO_4_/WO_3_ anodes were synthesized by a combination of glancing angle deposition, modified pulsed anodic electrodeposition, and spin coating. The microstructure of the MnO NP/BiVO_4_/WO_3_ anodes was studied using TEM. From these cross‐sectional TEM images, we also observed well‐aligned perpendicularly to the F:SnO_2_ (FTO) substrate and conformally coated BiVO_4_ nanodots on the entire surface of the WO_3_ nanorods without hindering the path of the electrolyte, as shown in **Figure** **2**a,b. We emphasize that pulsed electrodeposition facilitates the conformal deposition of an extremely thin BiVO_4_ layer on the entire surface of the WO_3_ nanorods. This can be also confirmed by energy dispersive spectrometer (EDS) images, as shown in Figure 2c–e. These results suggest that the overall active areas of the nanostructured BiVO_4_/WO_3_ heterojunction anodes are significantly increased. After the pulsed electrodeposition of the BiVO_4_ nanodots, 10 nm size MnO NPs were located on the top surface of the BiVO_4_/WO_3_ nanorods by using spin coating, as shown in Figure 2f. High‐resolution TEM (HR‐TEM) images and fast Fourier transform (FFT) pattern are shown in Figure 2h–k. The HR‐TEM images and electron diffraction patterns of the selected area showed *d*‐spacing of 0.398 and 0.312 nm, corresponding to the (102) and (112) planes of monoclinic BiVO_4_ nanodots, respectively, and *d*‐spacing of 0.311 nm, which can be assigned to the (110) plane of MnO NPs.

**Figure 3:**
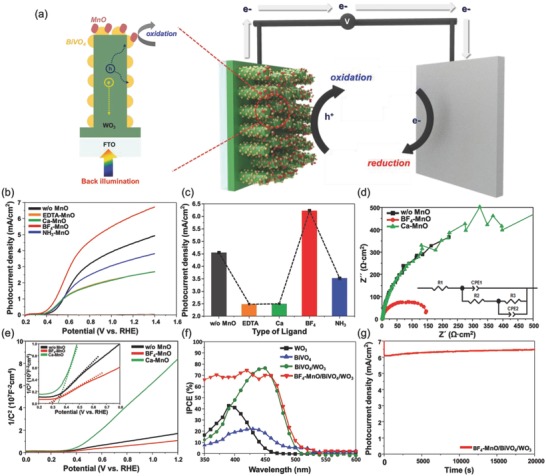
a) Schematic illustration of MnO NP/BiVO_4_/WO_3_ photoanodes under back‐side illumination and the operation of water splitting cell. b) Photocurrent density of MnO/BiVO_4_/WO_3_ anodes with diverse ligands of MnO. c) Comparison of photocurrent density at 1.23 V versus RHE with different ligands of MnO. d) Electrochemical impedance spectra for three types of BiVO_4_‐based anodes. e) Mott–Schottky plot of with BiVO_4_/WO_3_, BF_4_‐treated MnO/BIVO_4_/WO_3_, and Ca–EDTA‐treated MnO/BiVO_4_/WO_3_ measured under light off. The inset is enlarged Mott–Schottky plot (* frequency: 1 kHz, amplitude: 10 mV). f) IPCE spectra of WO_3_, BiVO_4_, BiVO_4_/WO_3_, and BF_4_‐treated MnO/BiVO_4_/WO_3_ at 1.23 V versus RHE. g) Stability test of BF_4_‐treated MnO/BiVO_4_/WO_3_ anode. The photoactivities are measured in presence of Na_2_SO_3_.

Four different ligands of MnO NPs (BF_4_, EDTA, Ca–EDTA, and NH_3_) were applied to prove the role of the ligand engineering of MnO NPs for enhancing water splitting efficiency. We compared the photocurrent density of MnO NPs with different ligands loaded on BiVO_4_/WO_3_ nanorods, as shown in **Figure** **3**. PEC measurements of the MnO/BiVO_4_/WO_3_ anodes with diverse ligands were performed using a standard three‐electrode cell with an electrolyte of 0.5 m phosphate buffer with 1 m sodium sulfite (Na_2_SO_3_) at a scan rate of 10 mV s^−1^ under 1.5 G solar light. The photo‐electrochemical properties of BiVO_4_‐based photoelectrodes were measured in the presence of 0.1 m Na_2_SO_3_, which served as the efficient hole scavenger. The oxidation of sulfite is thermodynamically and kinetically more facile than oxidation of water since the photogenerated holes are rapidly consumed for the oxidation of sulfite, thus measuring photocurrent in the sulfite oxidation enables investigation of the photo‐electrochemical properties of BiVO_4_‐based electrodes independently of its poor water oxidation kinetics. In the sulfite oxidation with extremely fast oxidation kinetics, in other words, Na_2_SO_3_ removing the injection barrier without affection the charge separation, surface recombination can be negligible. Therefore, most of the previously reported results related to BiVO_4_‐based photoanodes^13^, ^14^, ^16^, ^19^, ^47^, ^51^, ^52^, ^53^ were measured in sulfite oxidation condition to show photo‐electrochemical properties of BiVO_4_‐based electrodes independently of its poor water oxidation kinetics, as shown in Table S2 (Supporting Information). The photo‐electrochemical current densities of the BiVO_4_‐based photoanodes^4^, ^13^, ^14^, ^16^, ^17^, ^19^, ^20^, ^21^, ^29^, ^30^, ^31^, ^32^, ^33^, ^34^, ^35^, ^36^, ^37^, ^38^, ^39^, ^40^, ^41^, ^42^, ^43^, ^44^, ^45^, ^46^, ^47^, ^48^, ^49^, ^50^, ^51^, ^52^, ^53^, ^54^, ^55^, ^56^, ^57^, ^58^, ^59^, ^60^, ^61^, ^62^, ^64^, ^65^, ^66^, ^67^, ^68^, ^69^, ^77^ were plotted as a function of potential versus RHE. Thus, we measured PEC properties of BiVO_4_‐based anodes under sulfite oxidation to figure out the effect of ligand engineering. And, we compared photocurrent density of several anodes at the 1.23 V versus RHE since it is the theoretical minimum voltage for splitting water to oxygen and hydrogen.

We used the same concentration of MnO ligands (≈2 C) to exclude the coverage effect. The schematic illustration of the MnO NP/BiVO_4_/WO_3_ nanorods under back‐side illumination and the operation of the water splitting cell are shown in Figure 3a. Most of the light absorption occurs at the BiVO_4_/WO_3_ nanorods for solar water oxidation. And, the principal role of MnO NPs is the surface catalyst for reducing the rate of the electron–hole recombination by surface state passivation. And, it is possible to lower reaction overpotential and improve reaction kinetics by attaching the ligand‐engineered MnO NPs on the BiVO_4_/WO_3_ surface.^26^ Among the various types of ligands, BF_4_‐treated MnO NPs/BiVO_4_/WO_3_ show a drastically enhanced photocurrent density of about 1.4‐fold at the 1.23 V versus RHE, compared to the BiVO_4_/WO_3_ anode. However, the photoactivity of MnO NPs/BiVO_4_/WO_3_ with other ligands significantly decreased, as shown in Figure 3b. These results also revealed that the onset potential negatively shifted after the attachment of the MnO NPs. This result specifies that the introduction of MnO NPs leads to catalytic enhancement (reduction of overpotential), since an electrochemical cocatalyst could significantly facilitate the transfer of photogenerated holes from the semiconductors to water at a lower bias. Figure 3c shows the photocurrent behavior of the MnO NP/BiVO_4_/WO_3_ anodes with different ligands of MnO such as BF_4_, EDTA, Ca–EDTA, and NH_3_ at 1.23 V versus RHE. The BF_4_‐treated MnO/BiVO_4_/WO_3_ anode achieved 6.25 mA cm^−2^ at the 1.23 V versus RHE. We emphasize that the photocurrent of BF_4_‐treated MnO attached to the BiVO_4_/WO_3_ anode is the highest of the previously reported BiVO_4_‐based photoanodes with hole scavenger for solar water splitting, as shown in Table S2 and Figure S4 (Supporting Information).^13^, ^14^, ^16^, ^17^, ^21^, ^59^, ^60^, ^61^, ^62^, ^63^, ^64^, ^65^, ^66^, ^67^, ^68^, ^69^ Otherwise, Ca–EDTA‐treated MnO NPs/BiVO_4_/WO_3_ recorded the lowest photocurrent density of about 2.5 mA cm^−2^. Our results indicate that if the ligand exchange of MnO NPs is not conducted properly, it is difficult to observe the PEC enhancement of the BiVO_4_‐based anodes. The PEC properties of the MnO NP/BiVO_4_/WO_3_ anodes are considerably influenced by the variation of the ligand coordination environment and the ligand electron donating/withdrawing character.

We also compared photocurrent density of our final architecture as BF_4_–MnO/BiVO_4_/WO_3_ and Ca–EDTA–MnO/BiVO_4_/WO_3_ anodes with and without Na_2_SO_3_, respectively, as shown in Figure S5 (Supporting Information). In the low potential region, the photocurrent density without hole scavenger such as Na_2_SO_3_ is particularly low, indicating that the majority of the surface‐reaching holes were lost to surface recombination because of the poor catalytic nature of the BiVO_4_ surface for water oxidation. The oxidation of sulfite is thermodynamically and kinetically more facile than that of the oxidation of water, and thus, measuring photocurrent for sulfite oxidation enables investigation of the photo‐electrochemical properties of BiVO_4_‐based electrodes independent of its poor water oxidation kinetics.^21^ Thus, the superior photo‐electrochemical properties of BF_4_–MnO/BiVO_4_/WO_3_ in sulfite condition have enough potential in adjusting the water splitting system when the stubborn properties such as severe surface recombination of BiVO_4_ is solved after additional treatments.

Generally, several researches have studied the influence of surface chemistry on the PEC properties of various materials.^51^, ^52^, ^53^, ^54^ Surface chemistry modification using the ligand exchange technique can shift the ionization energy and band edge position of MnO NPs. Changing the identity of the chemical binding group and dipole moment of the ligands can also change the strength of the MnO–ligand surface dipole, shifting the vacuum energy and, in turn the MnO VBM and CBM. The modification of the surface chemistry related to the ligand exchange has typically been used to effectively passivate surface states and electronically couple with MnO NPs through decreasing the inter‐MnO NP distance, thus leading to enhanced carrier transport in MnO NPs and improved photoactivities.^50^, ^51^, ^52^, ^54^

To understand the effect of the ligand engineering of MnO NPs on PEC properties, we also compared the electrochemical impedance spectroscopy (EIS) of about 3 types of samples, bare BiVO_4_/WO_3_, BF_4_‐treated MnO, and Ca–EDTA‐treated MnO on BiVO_4_/WO_3_ anodes to evaluate the kinetics of charge generation, transfer, and separation during the OER. The impedance spectra of three types of BiVO_4_‐based photoanodes were measured by applying 1.23 V versus RHE under simulated solar light illumination, and are presented as a Nyquist diagram in the frequency range of 100 kHz–100 mHz. Generally, the Randles circuit composed by two resistance (series resistance and charge transfer resistance at the bulk) and one capacitance is used to analyze the EIS plot with one semicircle. It is difficult to fit our photoelectrode structure into the Randles circuit, since our MnO/BiVO_4_/WO_3_ photoelectrodes are comprised of the four interfaces, thus several resistance and capacitance are needed to analyze the EIS result. We used the Hamann equivalent circuit composed by 3 resistance and 2 capacitance to interpret our EIS plots. The impedance spectra of the complicated structure consisted of three arcs, they indicate the resistance of electrolyte, charge transfer, and mass transport, and three semicircles can be combined as one semicircle.^70^, ^71^, ^72^, ^73^ In the EIS circuit, *R*_s_ is the series resistance, *C*_s_ and *C*_ct_ are the constant phase element (CPE) for semiconductor interface and the electrolyte/electrode interface, respectively. R﻿﻿_ct1_ and *R*_ct2_ are the charge transfer resistances across the semiconductor interface and electrode/electrolyte interface, respectively. The measured data were fitted to the equivalent circuit, as shown in the inset of Figure 3d. The series resistance, charge transfer resistance, and capacitance obtained from the fittings, are summarized in Table S3 (Supporting Information). The high photoactivity is reflected by a small semicircle in the Nyquist plot. The fitted values of *R*_ct1_ are 22.36, 3.09, and 953.80 Ω cm^−2^ for the BiVO_4_/WO_3_, BF_4_–MnO/BiVO_4_/WO_3_, and Ca–MnO/BiVO_4_/WO_3_ anodes, respectively. Also, *R*_ct2_ is 898.11, 129.06, and 134.90 for the BiVO_4_/WO_3_, BF_4_–MnO/BiVO_4_/WO_3_, and Ca–MnO/BiVO_4_/WO_3_ photoanodes, respectively. Compared to anodes without MnO NPs, the charge transfer resistance of BF_4_‐treated MnO NPs loaded on the BiVO_4_/WO_3_ anode drastically decreased. The smallest semicircle, in the spectrum of the BF_4_‐treated MnO/BiVO_4_/WO_3_ photoanode, represents the fastest charge transport and indicates the effective charge transfer at the semiconductor interfaces and suppressed the charge recombination between the semiconductor and electrolyte. However, in case of the Ca–EDTA‐treated MnO loaded on the BiVO_4_/WO_3_ anode, it showed a substantially large charge transfer resistance between semiconductor interfaces. This indicates that it is significantly difficult to transfer the generated electron and hole pairs without recombination. This issue is closely related to the decline of the PEC efficiency of the Ca–EDTA‐ treated MnO NPs, as shown in Figure 3c. To verify the catalytic effect of MnO NPs, we compared EIS with pristine BiVO_4_, as shown in Figure S6 (Supporting Information). Charge transfer resistance (*R*_ct_) at the semiconductor interface and semiconductor/electrolyte interface significantly decreased after introduction of ligand‐engineered MnO NPs. We also measured EIS spectra of our final architectures such as BF_4_‐MnO/BiVO_4_/WO_3_ and Ca–EDTA–MnO/BiVO_4_/WO_3_ anodes with and without Na_2_SO_3_, respectively, as shown in Figure S7 and Table S3 (Supporting Information). Compared with sulfite oxidation, the charge transfer resistance in the semiconductor interface and semiconductor/electrolyte interface (*R*_ct1_ and *R*_ct2_) considerably increased under the water oxidation as without Na_2_SO_3_, since Na_2_SO_3_ removes the injection barrier without affecting the charge separation, and surface recombination of BiVO_4_ can be negligible.

We conducted the donor concentration, capacitance of the double layer, and flat band potential of without MnO NPs, BF_4_‐treated and Ca‐treated MnO NP–loaded BiVO_4_/WO_3_ using the Mott–Schottky (MS) relation, as displayed in Figure 3e. The Mott–Schottky method was used to investigate donor concentration and flat band potential of the electrodes (assuming flat photoelectrodes) by measuring the capacitance of the space charge region formed at the semiconductor/electrolyte interface at a fixed frequency of 1 kHz using the following equation

(1) $$C^{-2}=(2\;/\;e\varepsilon \varepsilon_{0}N_{D})\left[E-E_{\mathrm{fb}}-kT\;/\;e\right]$$

in which *C* = capacitance of space charge layer, *e* = electron charge (1.602 × 10^−19^ C), ε = dielectric constant, ε_0_ = permittivity of vacuum (8.854 × 10^−12^ F m^−1^), *V* (vs RHE) = applied potential, *V*_fb_ (vs RHE) = flat band potential (vs RHE), *k*_B_ = Boltzmann constant (1.381 × 10^−23^ J K^−1^), *T* = temperature (298 K), and *E* = applied potential. Some assumptions are made in the derivation of the Mott–Schottky equation; thus a standard nonlinear relationship between 1/*C* ^2^ and *V* may not be observed if such assumptions are not fulfilled (i.e., the space‐charge region capacitance being much less than the Helmholtz layer capacitance, a flat surface, absence of surface states, frequency independence of the dielectric constant, and homogeneous spatial distribution of donors/acceptors). Since our photoelectrodes have complicated structures, some assumptions to calculate Mott–Schottky equation are not maintained. Mott–Schottky plot for porous metal oxide can be dubious because the surface area of porous material is hard to confine. Also, the effect of double layer formed on metal oxide surface varies depending on electrolyte and applied frequency for measurement, thus it affects flat band potential. Even though Mott–Schottky plot does not provide absolute value, the tendency and reproducibility of the data make it reasonable to compare electrochemical properties of the several MnO/BiVO_4_/WO_3_ photoelectrodes. We measured Mott–Schottky plots at the same frequency of 1 kHz to grasp the tendency of MnO/BiVO_4_/WO_3_ photoelectrodes. The carrier concentration can be calculated from the slope of the Mott–Schottky curves. As the slope of Mott–Schottky plot flattens, the carrier concentration increases. The carrier concentration significantly increased after attachment of the BF_4_‐treated MnO NPs, while it decreased after attachment of Ca–EDTA‐treated MnO NPs. The general increment of *C* indicated that the double layer formed on the surface has a large capacitance.^66^ Increase in the capacitance caused the reduction of the depletion layer. The observed results (for 1/*C* ^2^ value, Ca–EDTA‐treated MnO NPs/BiVO_4_/WO_3_ > BiVO_4_/WO_3_ > BF_4_‐treated MnO NPs/BiVO_4_/WO_3_) were measured at the same frequency of 1 kHz. It is associated with highly capacitive ligand‐engineered BF_4_–MnO that can transfer photogenerated holes to the electrolyte.

The flat band potentials (*E*_fb_) were determined from the intercepts of 1/*C* ^2^ versus *V* subtracting *k*_B_ *T*/*e* = 0.025 V from the intercept. The charge carrier (donor) density (*N*_D_) is calculated from the slope of the 1/*C* ^2^ versus *V* curve using the following equation

(2) $$N_{D}=\left(2\;/\;\varepsilon \varepsilon_{0}e\right){\left[d\left(1\;/\;C^{2}\right)\;/\;d\left(V\right)\right]}^{-1}$$

The flat band potential is one of the key parameters to evaluate of the PEC performance when it is employed to drive electrochemical processes, such as water splitting. The observed onset potential deviates from the flat band potential severs as one of the measures of the effectiveness of the photoelectrodes. The flat band potential is determined experimentally using electrochemical and photo‐electrochemical methods when the semiconductor is immersed in the electrolyte solution. Unfortunately, the flat band potential cannot be measured directly; it is determined indirectly by fitting certain parameters, measurable on the electrode potential scale, to models of the semiconductor/electrolyte interface. Even though it is hard to observe exact flat band potentials, we can confirm the tendency of photo‐electrochemical properties. The flat band potential of BiVO_4_/WO_3_ is determined to be about 0.3 V versus RHE according to the Equation (2). After attaching the BF_4_‐treated MnO NPs, the *E*_fb_ shifts negatively by 30 mV, which is favorable for the electrons to pass through the circuit to the counter electrode. This is also a possible reason for the cathodic shift of the onset potential of the photocurrent, as shown in Figure 3a. Unlikely, the Ca–EDTA‐treated MnO NPs loaded on BiVO_4_/WO_3_ showed a positive shift by 50 mV.

The incident photon to current conversion efficiency (IPCE) is conducted to understand the interplay between the photoactivity and the light absorption of the WO_3_, BiVO_4_, BiVO_4_/WO_3_, and BF_4_‐treated MnO NPs/BiVO_4_/WO_3_, as a function of the wavelength of the incident light. IPCE spectra were measured from 350 to 600 nm at 1.23 V versus RHE, as presented in Figure 3f. BF_4_‐treated MnO attached to the BiVO_4_/WO_3_ anode exhibits a higher IPCE value over the entire optical region than without MnO NPs. The photoresponse extended to 530 nm and the intensity was substantially increased up to 75% at the low wavelength region. The photocurrent density was measured at 1.23 V versus RHE continuously for 20 000 s under AM 1.5 G condition to determine the stability of the BF_4_‐treated MnO–loaded BiVO_4_/WO_3_ anode, as shown in Figure 3g. The photocurrent density of 6.2 ± 0.1 mA cm^−2^ was consistent for 20 000 s, demonstrating the potential of BF_4_‐treated MnO/BiVO_4_/WO_3_ as photoanodes for practical applications. We measured photocurrent density of BF_4_‐treated MnO/BiVO_4_/WO_3_ synthesized a year ago, and obtained similar properties as the as‐prepared, as shown in Figure S8 (Supporting Information). This can be an evidence for existence of ligands, since nanoparticles without ligands can be easily agglomerated during reaction, they block the gap between the WO_3_ nanorods, and thus deteriorate the photoactivities.

To compare the coverage effect of BF_4_‐treated MnO NPs on BiVO_4_/WO_3_ photoanodes, we controlled the concentration of BF_4_‐treated MnO NPs to adjust the amount of MnO NPs on the BiVO_4_/WO_3_ anodes. We measured the PEC properties of the BF_4_‐treated MnO/BiVO_4_/WO_3_ anodes with different coverages of BF_4_‐treated MnO NPs. The increased coverage of catalytic MnO NPs boosts charge transfer and surface catalytic properties, and thus improve photoactivities. However, when the concentration of BF_4_‐treated MnO NPs is more than 2 C, the photocurrent density of BF_4_‐treated MnO/BiVO_4_/WO_3_ decreases gradually with the increase in concentration, as shown in Figure S9 (Supporting Information). These results also indicate that the onset potential of the 4 C MnO NPs loaded on BiVO_4_/WO_3_ positively shifted compared to that of the BiVO_4_/WO_3_ anode. This photocurrent degradation could be caused by two possible reasons. First, MnO NPs are agglomerated when the concentration of MnO exceeded the specific value and may cover a wide portion of the BiVO_4_/WO_3_ surface, thus can block the gap between the WO_3_ nanorods, as shown in Figure S10 (Supporting Information). It is significantly hard to transfer of the generated electron and hole pairs in the BiVO_4_ and WO_3_ to the electrolyte for water oxidation reaction. The agglomerated MnO NPs can obstruct sites of electrolyte permeation, retard the light absorption, and thus impede the charge transfer for water oxidation, as shown in Figure S11 (Supporting Information).^74^ Since the generated charges in the BiVO_4_ have to transfer via path 1) with long distance rather than path 2) with short distance reaction to interact with electrolyte for oxygen evolution, it cause the charge recombination, and then increases the charge transfer resistance at the semiconductor interface. In other words, the high coverage of the MnO NPs may reduce the surface area of BiVO_4_/WO_3_ that is in direct contact with the electrolyte, thereby hindering the water oxidation performance. It can be confirmed by EIS data, as shown in Figure S9f,g and Table S4 (Supporting Information). The charge transfer resistance at the semiconductor interface (*R*_ct1_) decreases gradually until the concentration of MnO NPs increases to 2 C by 3.09 Ω cm^−2^, however the resistance then starts to drastically increase up to 852 Ω cm^−2^ after loading of 4 C MnO NPs. The photocurrent density of BF_4_‐treated MnO/BiVO_4_/WO_3_ having the concentration of 4 C recorded a lower value than that the BiVO_4_/WO_3_ anode due to the severe charge recombination at the interface. Second reason is poor catalytic properties of 4 C MnO NPs at the surface for water oxidation. Beyond the optimum concentration of MnO NPs, the charge transfer properties at the electrode/electrolyte are considerably declined since charges are hard to participate to water oxidation due to the recombination. It is supported by resistance data, the charge transfer resistance at the electrode/electrolyte interface (*R*_ct2_) is slightly increased in the 4 C MnO loaded on BiVO_4_/WO_3_ anode. The highest photocurrent of 6.25 mA cm^−2^ was obtained at 1.23 V versus RHE for BF_4_‐treated MnO/BiVO_4_/WO_3_ with 2 C concentration of MnO NPs. From these results, we believe that the amounts of attached MnO NPs must be properly controlled, since a high coverage of MnO NPs on the semiconductor will reduce the active areas of the semiconductor, hindering the reactant access of the semiconductor, while low coverage will lead to poor light utilization efficiency during photocatalysis. We also confirmed the good reproducibility of BF_4_‐treated MnO NPs/BiVO_4_/WO_3_, as shown in Figure S9h (Supporting Information). Most of the BF_4_‐treated MnO NPs/BiVO_4_/WO_3_ showed a similar photocurrent density, clarifying that our photoelectrodes show potential for practical application. Since the synthesis and attachment methods of MnO NPs are similar regardless of the ligands, the reproducibility is obtained even in case of the other ligand of MnO.

**Figure 4:**
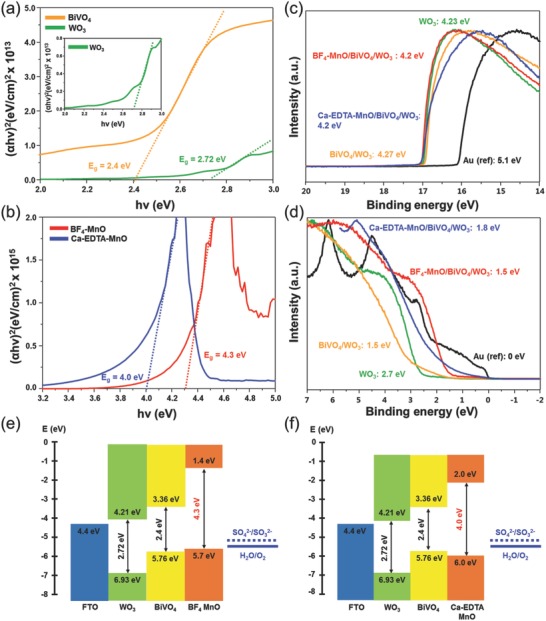
a,b) UV–vis absorption spectra of WO_3_, BiVO_4_, BF_4_‐treated MnO, Ca–EDTA‐treated MnO. c) The secondary electron emission spectra of the WO_3_, BiVO_4_/WO_3_, BF_4_‐treated MnO/BiVO_4_/WO_3_, Ca‐treated MnO/BiVO_4_/WO_3_, and reference Au foil electrodes. d) Valence band spectra, the energy difference between the Fermi level and the valence band maximum (*E*_F_ − *E*_V_) of WO_3_, BiVO_4_/WO_3_, BF_4_‐treated MnO/BiVO_4_/WO_3_, Ca‐treated MnO/BiVO_4_/WO_3_. e,f) Flat band structure of BF_4_‐treated MnO/BiVO_4_/WO_3_, Ca‐treated MnO/BiVO_4_/WO_3_, respectively.

We believe that the discrepancy in the photoactivities between BF_4_‐treated MnO and Ca–EDTA‐treated MnO NPs attached to BiVO_4_/WO_3_ anodes is attributed to the considerable alteration of the band structure through the ligand engineering of MnO NPs. UV–vis spectroscopy and ultraviolet photoelectron spectroscopy (UPS) measurements were performed to investigate the modified band structure of MnO/BiVO_4_/WO_3_ with different ligands. The UPS measures occupied electronic states and thus provides information on the Fermi level (low‐binding‐energy cutoff) and VBM of a material. The energy *E*_C_ of CBM can be approximated by adding the electronic transport gap *E*_g_ of the material to the VBM, where *E*_g_ is determined from the sum of the optical bandgap. UV–vis spectroscopy gives an idea about the optical bandgap energy and thus provides an important clue for investigation. The energy band structure of a photoelectrodes is considered to be a key factor in determining their photocatalytic activity. The optical bandgap of WO_3_ and BiVO_4_ is 2.72 and 2.4 eV, respectively, as shown in **Figure** **4**a. We also measured the bandgaps of BF_4_‐treated MnO and Ca–EDTA‐treated MnO, which are 4.3 and 4.0 eV, respectively, as shown in Figure 4b. The bandgap can be evaluated from the following equation of

(3) $$\alpha \left(hv\right)=A{\left(hv-E_{g}\right)}^{n/2}$$

in which α, ν, *E*_g_, and *A* are the absorption coefficient, light frequency, bandgap energy, and a constant, respectively.

In addition, the absorption coefficient of WO_3_, BiVO_4_, BF_4_‐treated MnO, and Ca–EDTA‐treated MnO is measured, as shown in Figure S12 (Supporting Information). The values determined in this measurement are in good agreement with those reported in the literature.^17^, ^75^ The secondary electron emission spectra (SEE) of the WO_3_, BiVO_4_/WO_3_, BF_4_‐treated MnO/BiVO_4_/WO_3_, Ca–EDTA‐treated MnO/BiVO_4_/WO_3_, and reference Au foil electrodes are displayed in Figure 4c. Compared with the work function of the Au reference of 5.1 eV, the work functions of the sample can be estimated from the SEE cutoffs as 4.23, 4.27, 4.2, and 4.2 eV for the WO_3_, BiVO_4_/WO_3_, BF_4_‐treated MnO/BiVO_4_/WO_3_, and Ca–EDTA‐treated MnO/BiVO_4_/WO_3_, respectively. According to the valence band spectra, the energy difference between the Fermi level and the valence band maximum (*E*_F_ − *E*_V_) for WO_3_ and BiVO_4_/WO_3_ is 2.7 and 1.5 eV, respectively. The *E*_F_ − *E*_V_ for the BF_4_‐treated MnO/BiVO_4_/WO_3_ and Ca‐treated MnO/BiVO_4_/WO_3_ is 1.5 and 1.8 eV, as shown in Figure 4d. The values measured by UPS are summarized in Table S5 (Supporting Information). Based on these results, the energy band diagram for the BF_4_‐treated MnO/BiVO_4_/WO_3_ and Ca–EDTA‐treated MnO/BiVO_4_/WO_3_ is illustrated in Figure 4e,f. The band structures clearly indicate that the difference of photoactivities between BF_4_‐treated MnO/BiVO_4_/WO_3_ and Ca–EDTA‐treated MnO/BiVO_4_/WO_3_ is attributed to the valence band position of MnO with different ligands. The BiVO_4_/WO_3_ heterojunction anodes are widely known as the type II structure, whereby it is possible to transfer and separate the generated charge carriers without recombination at the interface. The band structure of our BiVO_4_/WO_3_ anodes also indicated type II junction; the band position of MnO has critical impact on the enhanced photoactivities of MnO/BiVO_4_/WO_3_ anodes. The bandgap of MnO is slightly modified according to the ligand engineering, thus VBM and CBM shift to match the energy difference, indicating that the band edge shifts are electrostatic in origin. The VBM of BF_4_‐treated MnO is slightly higher than that of BiVO_4_, while Ca–EDTA‐treated MnO is positioned considerably lower than that of BiVO_4_. The band diagram, as shown in Figure 4e clearly shows that the transport of the photogenerated electrons and holes from WO_3_ to BF_4_‐treated MnO is energetically favorable. Otherwise, the VBM of Ca–EDTA‐treated MnO is lower than that of BiVO_4_, thus photogenerated holes are difficult to transfer from BiVO_4_ to Ca–EDTA‐treated MnO for water oxidation. This result also closely corresponds to the resistance data from the EIS measurement, as displayed in Figure 3d and Table S3 (Supporting Information). The charge transfer resistance between the semiconductors (*R*_ct1_) of the Ca–EDTA‐treated MnO/BiVO_4_/WO_3_ anodes is significantly higher than the BF_4_‐treated MnO/BiVO_4_/WO_3_ anodes.

We also analyzed UPS spectra of untreated MnO/BiVO_4_/WO_3_ to verify the effect of ligand engineering, as shown in Figure S13 (Supporting Information). The optical bandgap of commercialized MnO is 4.13 eV, as shown in Figure S13a (Supporting Information). The SEE of the untreated MnO/BiVO_4_/WO_3_ and reference Au foil electrodes are displayed in Figure S13b (Supporting Information). Compared with the work function of the Au reference of 5.1 eV, the work function of the untreated MnO/BiVO_4_/WO_3_ is 4.26 eV. According to the valence band spectra, the *E*_F_ − *E*_V_ for the untreated MnO/BiVO_4_/WO_3_ is 2.26 eV, as shown in Figure S13c (Supporting Information). The values measured by UPS are summarized in Table S5 (Supporting Information). Based on these results, the energy band diagram for the untreated MnO/BiVO_4_/WO_3_ is illustrated in Figure S13d (Supporting Information). The VBM of untreated MnO is positioned considerably lower than that of BiVO_4_, thus it is hard to transfer of photogenerated holes from BiVO_4_ to untreated MnO for water oxidation. From these results, we realized that it is necessary to introduce ligand engineering to adjust MnO NPs as an efficient oxygen evolution catalyst for BiVO_4_‐based cascade photoanodes.

The shift in the band edges of MnO upon ligand adsorption can be conceptualized as the sum of two dipole contributions: a contribution from the dipole formed between the surface atom of the MnO and the binding group of the ligand, associated with structural distortion and charge rearrangement, and a contribution from the intrinsic dipole moment of the ligand itself. By altering the binding group, induced dipole, and orientation of the ligand's intrinsic dipole moment via directed functionalization strategies, it is possible to tune the band edge energies for the optimal water oxidation. The significant shift of the band edge energies predicted is electrostatic in origin and results from a dipole layer at the surface associated with the chemisorbed ligands. The shift in the electron and hole energy is aligned with the effective dipole density at the surface. Generally, the induced dipole moment is fixed by the chemical interaction between the ligand and the surface; it is difficult to modify the band edge energies without altering the nature of the bonding. On the other hand, altering the intrinsic dipole moment through judicious functionalization offers a controllable way to tune band energies, particularly for a stable chemical bond between the ligand and surface. The shifts of the band edge energies of MnO can still be tuned over a wide range by controlling the intrinsic dipole moment of the ligand. Since the orientation and coverage of the ligands on the surface, and the contribution of the effective dipole moment are weakly coupled, we can predict the change in the energy shift from the controlled change in the intrinsic dipole moment of the ligand.^50^, ^51^, ^52^, ^53^, ^54^

Another possible reason for the disparity of photoactivities between the BF_4_‐treated MnO and Ca–EDTA‐treated MnO is the presence of fluorine. The fluorinated ligands are not likely to interdigitate due to dipolar fluorine–fluorine electrostatic interaction; thus, will exhibit band edge positions closer to those of the MnO. Since fluorine is the most electronegative element of the periodic table, MnO—F bonds are highly dipolar, inducing the fluorine atoms of interacting ligand to repel one another and prevent interdigitation.^54^ The specific research reveals that the interdigitization reduces the magnitude of the band shift.^54^ The substantial shift of the band edge induced by the surface ligand layer can modify the final band structure of the MnO/BiVO_4_/WO_3_ anodes. The difference in the energetic environment of the BiVO_4_/WO_3_ anodes with BF_4_‐treated MnO and Ca–EDTA‐treated MnO induces a shift of the energy levels of the MnO; thus, it enables to explain disparity of the photoactivities. Thus, with proper ligands, the energy level of MnO can be shifted toward energies where both the CBM and VBM straddle the hydrogen and oxygen redox potentials, which is ideal for water splitting.

**Figure 5:**
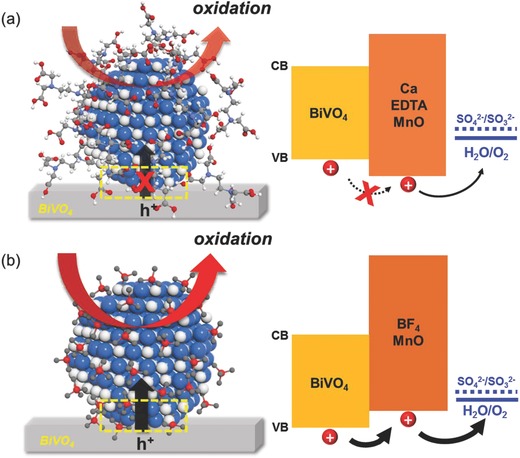
The structural formulas and band structure of a) Ca–EDTA‐treated MnO/BiVO_4_ and b) BF_4_‐treated MnO/BiVO_4_.

The optimized geometric factors such as the length of ligands have a crucial impact on the efficiency of solar water splitting, since controlling the length of the ligand is recognized as essential for efficient charge transfer; which is one of the most important criteria determining the photoactivities. The long organic ligands, which are used to control growth as well as to stabilize the host material, significantly inhibit the charge transfer at the interfacial region, leading to inefficient solar water splitting.^76^ To increase the charge transfer at the interface, shorter ligands need to be utilized. The carrier mobility decreases exponentially with the increasing of the ligand length, demonstrating the inverse relationship between coupling energy and inter‐MnO distance. Considering the molecular formula, as shown in Figure S14 (Supporting Information) and **Figure** **5**, it is reasonable that the length of the BF_4_ ligand is significantly shorter than that of the Ca–EDTA ligand. This is closely related to the values of the flat band potential of MnO/BiVO_4_/WO_3_ with different ligands and charge transfer resistances between the semiconductors, as shown in Figure 3d,e. This therefore reveals that reducing the distance between MnO with ligands and BiVO_4_ improves the charge transfer rate, leading to an increase in overall water oxidation efficiency.

We would like to highlight that the ligand engineering of catalytic nanoparticles on photoelectrodes is the critical factor for efficient water oxidation, since the band edge position is drastically shifted according to the type of ligand. The structural formula and band structure of MnO/BiVO_4_ electrodes with different ligands are shown in Figure 5. In case of the Ca–EDTA‐treated catalytic MnO NPs, the VBM and CBM are shifted downward, thus it is might be hard to transfer photogenerated holes from BiVO_4_ to MnO without loss due to the barrier at the interface. Otherwise, band edges of BF_4_‐treated MnO are suitably positioned to permit transfer of holes for forceful water oxidation. In these systems, BF_4_‐treated MnO NPs act as an efficient charge transfer mediator for PEC water splitting.

## 3 Conclusions

We studied the ligand engineering of MnO NPs to enhance the photoactivity of BiVO_4_‐based anodes, and found that BF_4_‐treated MnO/BiVO_4_/WO_3_ showed superior PEC properties to the Ca–EDTA‐treated MnO/BiVO_4_/WO_3_, due to the well‐aligned band edge position of the BF_4_‐treated MnO/BiVO_4_/WO_3_ photoelectrode. The BF_4_‐treated MnO/BiVO_4_/WO_3_ showed a markedly enhanced photocurrent density of 6.25 mA cm^−2^ at 1.23 V versus RHE in the presence of Na_2_SO_3_. The improved PEC performance of these photoelectrodes is due to their higher charge generation and separation rate through the modification of the band structure, which enables forceful water oxidation. The trends in the energy level position between the different ligands are confirmed by UPS measurement, showing the observed result from contributions from the both the MnO–ligand interface dipole and the intrinsic dipole moment of the ligand molecule itself. These energy level shifts result in predictable changes in the water splitting system and provide a guide to the optimal ligand choice and following band structure of anodes for photoactivities. These findings have recently been employed to guide the design of a photoelectrodes employing a cascaded energy level architecture. These results shed light on the clue that ligand‐induced band energy shift, in conjunction with quantum confinement–controlled bandgap modification, is a critical adjustable parameter in the optimization of the water splitting system.

## 4 Experimental Section

*Synthesis of BiVO_4_‐Based Heterojunction Anodes*: BiVO_4_‐based heterojunction anodes were synthesized by combination with glancing angle deposition (GLAD) and modified pulsed anodic electrodeposition. In this study, FTO substrate was 1.5 × 1.5 cm and the active site region (1.5 × 1.0 cm) was defined with a shadow mask. First, several metal oxide films and WO_3_ nanorods were formed using the GLAD technique. Metal oxide powder (Taewon Co.) was placed in a carbon crucible and subjected to e‐beam evaporation. Prior to WO_3_ nanorod fabrication, a thin WO_3_ film (≈50 nm) was deposited to improve adhesion and then the substrate was spun with various glancing angles to form WO_3_ nanorod arrays. The pressure in the chamber was maintained at 3.5 × 10^−6^ Torr and the rotation speed was 80 rpm. The as‐deposited samples were converted to crystalline phase by annealing in air at 500 °C for 2 h. Moreover, deposition at a glancing angle combined with a uniform rotation speed resulted in the formation of well‐separated vertical WO_3_ nanorods. Subsequently, BiVO_4_ was deposited on the WO_3_ nanorods by modified pulsed electrodeposition. Precursor was prepared by dissolving bismuth nitrate pentahydrate (BiN_3_O_9_, 98%, JUN) in a solution of 30 × 10^−3^ m vanadium oxide sulfate hydrate (VOSO_4_, 99.99%, Aldrich) at pH < 0.5 with nitric acid (HNO_3_, 67%, JUN). Then, 2 m sodium acetate (CH_3_COONa, Aldrich) was added, raising the pH to ≈5.1, which was then adjusted to pH 4.7 using a few drops of concentrated HNO_3_. This mildly acidic pH condition is necessary because at pH values of >5, V (IV) precipitates form in the solution. Pulsed anodic electrodeposition was conducted in a standard three‐electrode system with a working electrode of WO_3_ nanorods, a Ag/AgCl reference electrode, and a platinum counter electrode. Deposition of amorphous Bi–V–O was carried out potentiostatically at 1.95 V versus Ag/AgCl for 1 min at 80 °C (≈2–3 mA cm^−2^) and then all freshly prepared samples were rinsed and annealed at 500 °C for 6 h in air at a heating rate of 2 °C min^−1^. After annealing, the as‐deposited films were converted to a crystalline monoclinic phase of BiVO_4_. Unlike previous methods, pulsed electrodeposition enables conformal deposition on the bottom electrode, which affects the photo‐electrochemical property.

*MnO NP Synthesis and Ligand Exchange*: Typically, hot‐injection synthesis for MnO NPs required two separate reaction pots. First, 1 mmol of Mn(III) acetate and 2 mmol of myristic acid were dissolved in 20 mL of octadecene. The other reaction pot was comprised of 0.45 mL of decanol in 3 mL of octadecene. These two separate mixtures were degassed at 110 °C for 1 h to completely remove oxygen. The carboxylate mixture was heated up to 295 °C under argon atmosphere. Then, the mixture of decanol was injected into the carboxylate reaction pot and it was maintained at 285 °C for 1 h. After cooling down to room temperature, the dark brown solution was purified with acetone and toluene mixture. The as‐prepared MnO NPs were well dispersed in nonpolar solvent, hexane or octane. The MnO NP solution concentration used for ligand exchange could be about 1.5–2.0 mg mL^−1^. In this work, four types of ligand molecules were used for surface treatment: EDTA, Ca–EDTA, BF_4_, and NH_4_.

*EDTA–MnO NPs, Ca–EDTA‐decorated MnO NPs*: First, 0.01 m EDTA solution was prepared in methanol solution. To deprotonate the ligand, 4 equivalent trimethylamine was added in the solution and sonicated over 1 h. Then, the as‐prepared MnO NP in hexane solution was mixed with 0.01 m deprotonated EDTA–methanol solution at one‐to‐one volume ratio. The mixed solution was vortexed for 1 h. Subsequently, as a washing step, centrifugation was performed with excess amount of toluene for 3 times. These ligand exchange and purification steps were repeated 3 times. Finally, precipitated EDTA–MnO NPs were redispersed in polar solvents. To incorporate Ca^2+^, 0.01 m CaCl_2_ solution was additionally inserted in precipitated EDTA–MnO NPs and mixed for 1 h. After ligand exchange process, surface‐treated MnO NPs were spin‐coated on BiVO_4_/WO_3_ /FTO electrode. For convenience, when volume ratio of the initial MnO solution to polar solvent was 1, its concentration was called 1 C. The concentration of MnO NPs was marked as C, meaning that 1 C concentration meant 1 mg of MnO nanoparticles in 1 mL of ethanol solution. For example, if 10 µL of MnO solution was washed and dispersed in 10 µL of ethanol after surface treatment, concentration of final solution was defined as1 C.

*BF_4_^−^Decorated MnO NPs*: First, 0.01 m NOBF_4_ solution was prepared in dimethylformamide (DMF) solution. Then, the as‐prepared MnO NPs in hexane solution were mixed with 0.01 m NOBF_4_–DMF solution at one‐to‐one volume ratio. The mixed solution was vortexed for 30 min. Subsequently, as a washing step, centrifugation was performed with excess amount of toluene for 3 times. These ligand exchange and washing steps were repeated 3 times. Finally, precipitated BF_4_–MnO NPs were redispersed in polar solvents.

*NH_4_^+^*−*Treated MnO NPs*: The as‐prepared MnO NPs dispersed in hexane were spin‐coated on BiVO_4_/WO_3_/FTO electrode. Then, the electrode was dipped in 25 wt% NH_4_OH solution for 1 h. After dipping, the electrode was rinsed with DI water and dried in oven.

*PEC Measurement*: The photo‐electrochemical properties of BiVO_4_‐based photoelectrodes were measured in phosphate buffer solution with the presence of 0.1 m Na_2_SO_3_, which served as an efficient hole scavenger. The oxidation of sulfite is thermodynamically and kinetically more facile than the oxidation of water, since the photogenerated holes are rapidly consumed for the oxidation of sulfite, thus measuring photocurrent in the sulfite oxidation enables investigation of the photo‐electrochemical properties of BiVO_4_‐based electrodes independently of its poor water oxidation kinetics. The photocurrent versus potential curve was recorded while sweeping the potential in the positive direction with a scan rate of 10 mV s^−1^ under a solar simulator with an AM 1.5 G filter; the light intensity of the solar simulator was calibrated to 1 sun (100 mW cm^−2^), using a reference cell. The IPCE was measured using a light source and monochromator at 1.23 V versus RHE. The EIS was conducted by applying 1.23 V versus RHE. The sweeping frequency was from 100 kHz to 100 mHz, with an AC amplitude of 10 mV. The measured spectra were fitted by using the ZSimpWin software. The Mott–Schottky plot was measured with a frequency of 1 kHz under light off.

*Characterization*: The morphologies of BiVO_4_‐based anodes were characterized by field‐emission scanning electron microscopy (MERLIN Compact, JEOL). Bright‐field and high‐resolution transmission electron microscopy (JEM‐2100F, JEOL) with 200 kV field‐emission images were obtained to investigate the microstructure of the BiVO_4_‐based anodes. XRD characterization was performed to confirm the crystalline phase of MnO. The absorption spectra of the WO_3_, BiVO_4_, and MnO were measured by UV–visible spectroscopy (JASCO‐670). The band positions of BiVO_4_‐based anodes were determined by using the ultraviolet photoelectron spectroscopy (UPS). Fourier transform infrared spectroscopy and X‐ray photoelectron spectroscopy were also conducted to analyze ligand exchange of MnO.

## Conflict of Interest

The authors declare no conflict of interest.

## Supporting information

Supplementary
