# Supplementary material for: Efficient Water Splitting Cascade Photoanodes with Ligand‐Engineered MnO Cocatalysts
Source: Adv Sci (Weinh). 2018 Aug 6;5(10):1800727. doi: 10.1002/advs.201800727 (PMC6193156; doi:10.1002/advs.201800727)
Supplement: Supplementary file 1 — Supplementary [file ADVS-5-1800727-s001.pdf]

Copyright WILEY-VCH Verlag GmbH & Co. KGaA, 69469 Weinheim, Germany, 2018.

Supporting Information

for *Adv. Sci.,* DOI: 10.1002/advs.201800727

Efficient Water Splitting Cascade Photoanodes with Ligand-Engineered MnO Cocatalysts

Mi Gyoung Lee, Kyoungsuk Jin, Ki Chang Kwon, Woonbae Sohn, Hoonkee Park, Kyoung Soon Choi, Yoo Kyung Go, Hongmin Seo, Jung Sug Hong, Ki Tae Nam, and Ho Won Jang**

Copyright WILEY-VCH Verlag GmbH & Co. KGaA, 69469 Weinheim, Germany, 2018.

Supporting Information

Efficient Water Splitting Cascade Photoanodes with Ligand Engineered MnO Co-catalysts

Mi Gyoung Lee[‡a], Kyoungsuk Jin[‡a], Ki Chang Kwon[a], Woonbae Sohn[a], Hoonkee Park[a], Kyoung Soon Choi[b], Yoo Kyung Go[a], Hongmin Seo[a], Jung Sug Hong[a], Ki Tae Nam[a], Ho Won Jang*[a]*

[a]Department of Materials Science and Engineering, Research Institute of Advanced Materials, Seoul National University, Seoul 151-744, Republic of Korea

[b]Advanced Nano Surface Research Group, Korea Basic Science Institute, Daejeon 34133, Republic of Korea

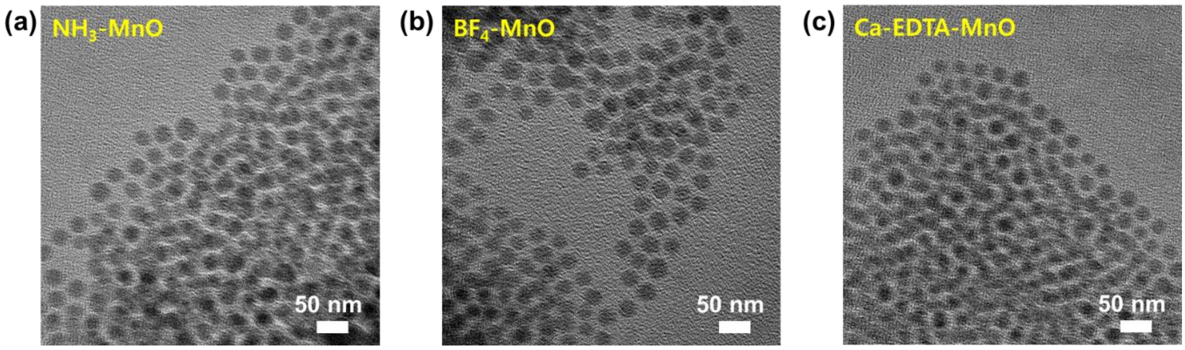

Figure S1. TEM images (a) NH_3-treated MnO NPs, (b) BF_4-treated MnO NPs and (c) Ca-EDTA-treated MnO NPs.

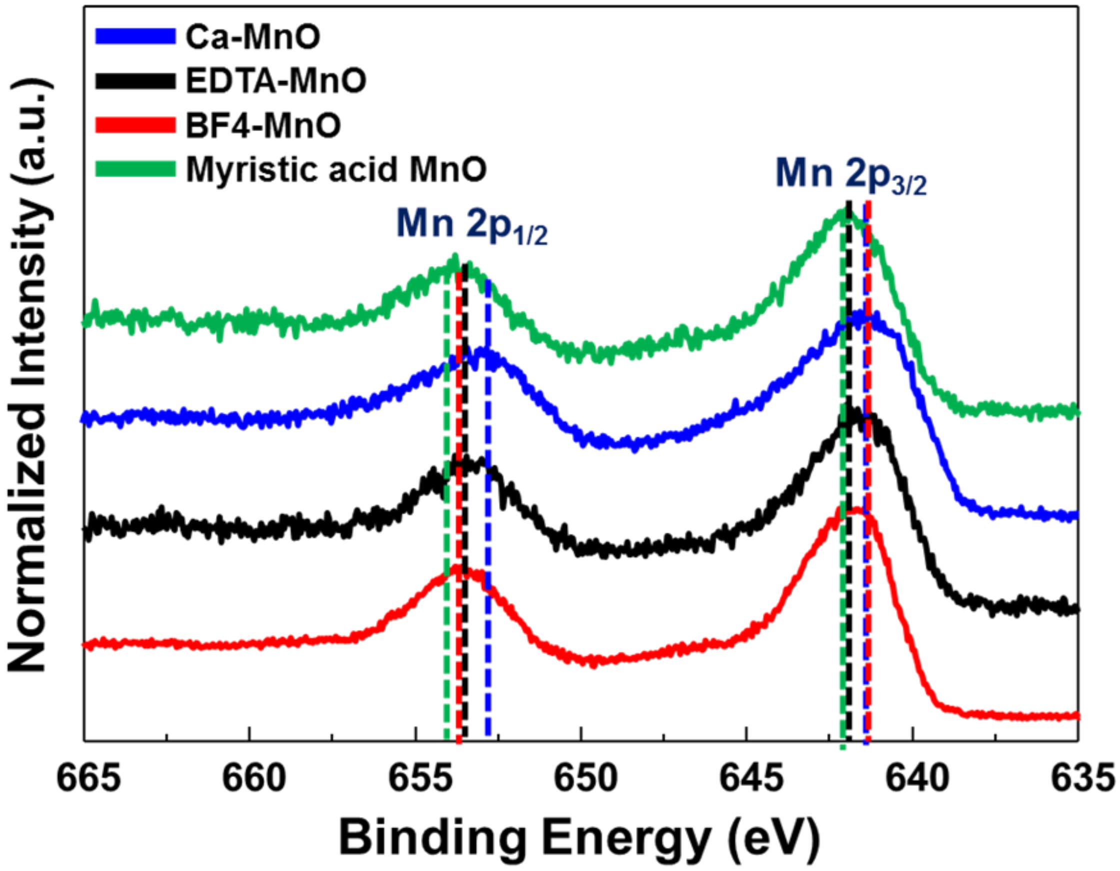

Figure S2. X-ray photoelectron spectroscopy (XPS) spectra of MnO with different ligands.

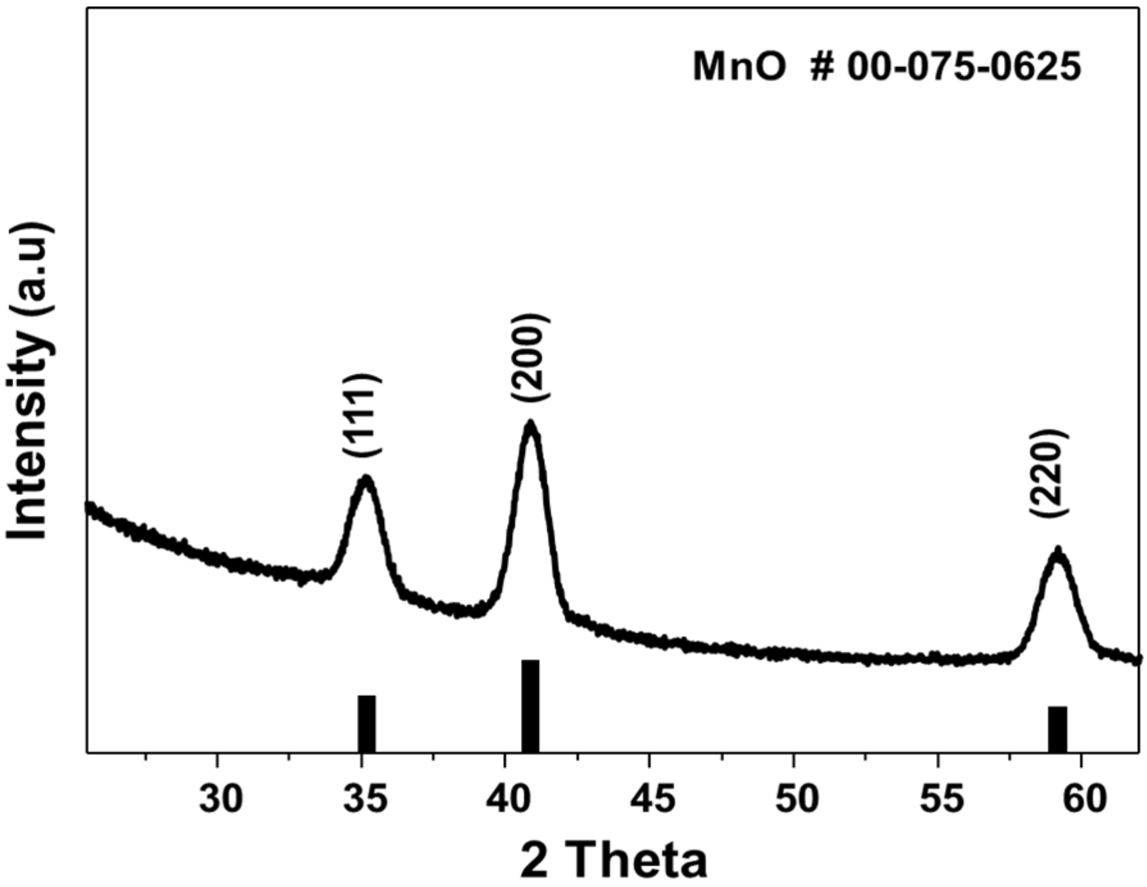

Figure S3. X-ray diffraction (XRD) pattern of MnO crystal.

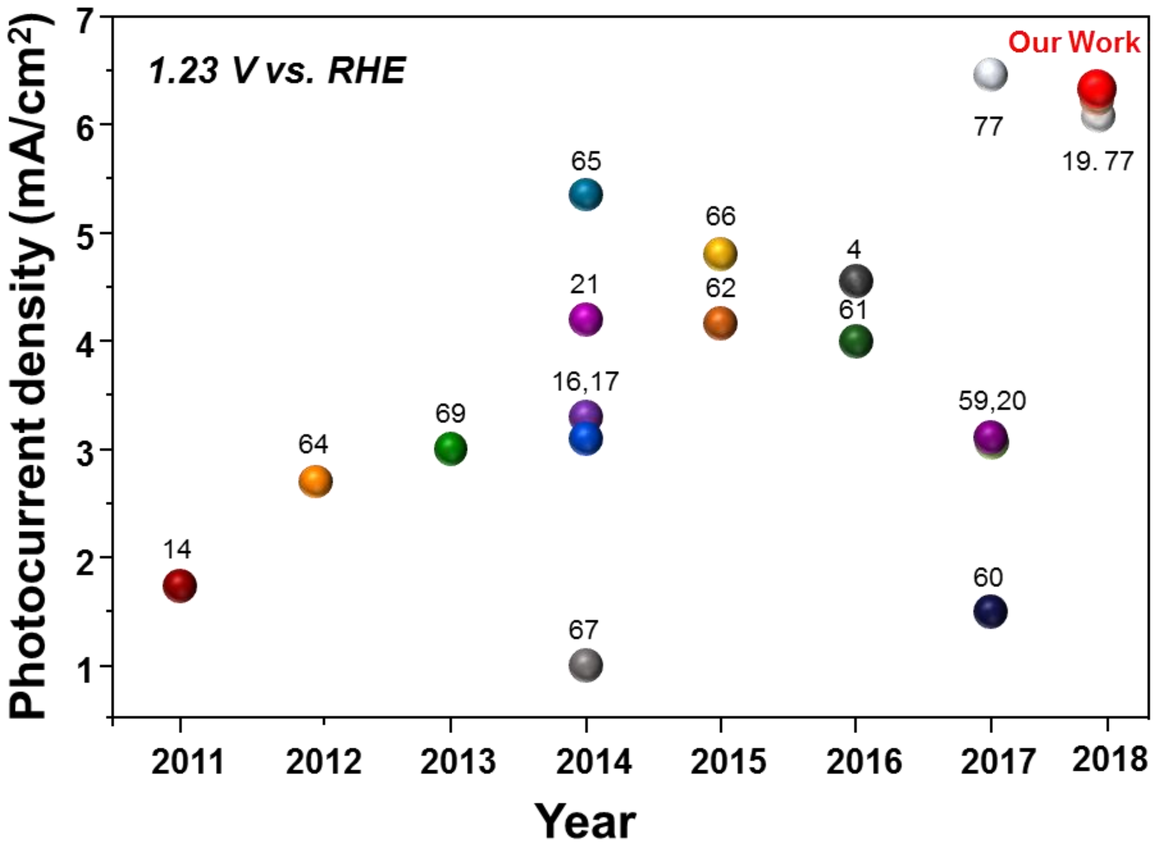

Figure S4. Summary of $BiVO_4$-based electrodes for PEC water splitting and their phoc urrent denisty at the 1.23 V vs. RHE in recent years (2011-2018).

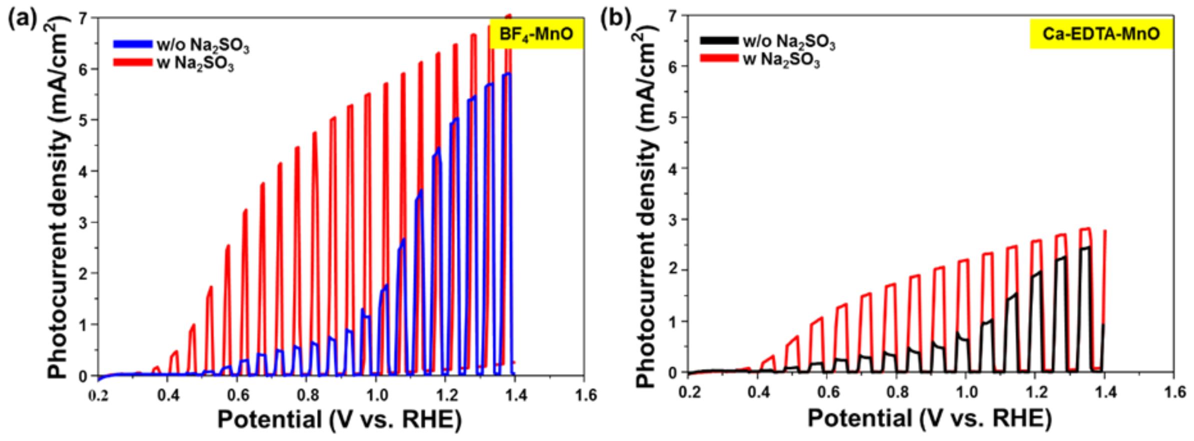

Figure S5. Linear sweep voltammograms for (a) BF_4-MnO/$BiVO_4$/WO_3, and (b) Ca-EDTA-MnO/$BiVO_4$/WO_3 anodes measured with and without Na_2OS_3 at a scan rate of 10mV/s under 1.5 G solar light under the chopped light condition.

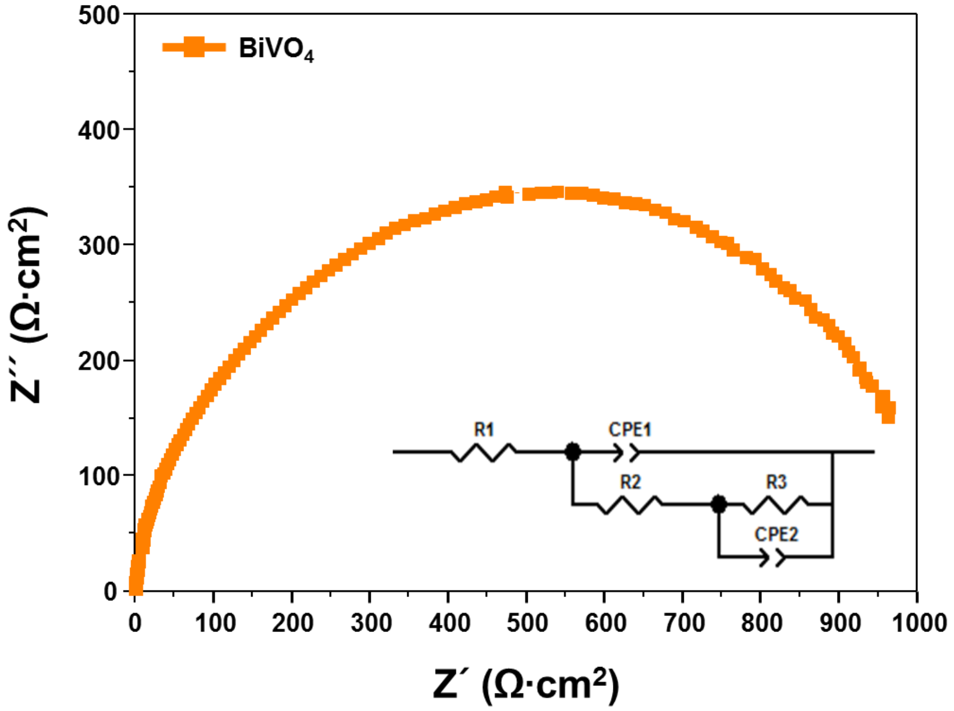

Figure S6. Electrochemical impedance spectra for pristine $BiVO_4$.

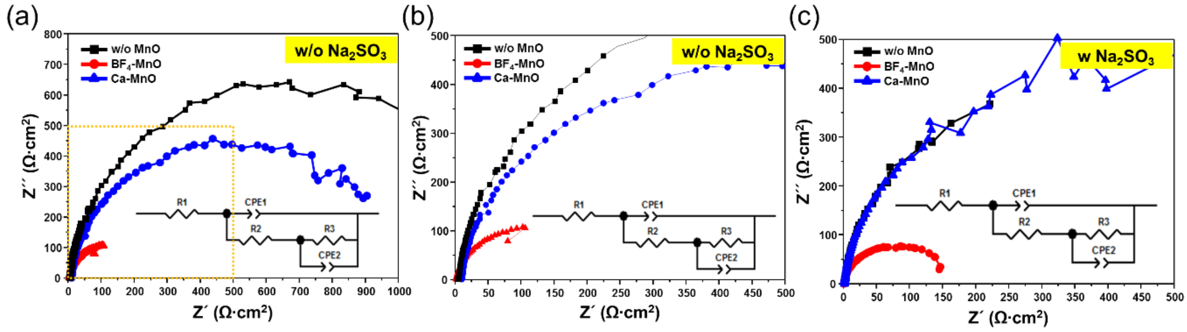

Figure S7. Electrochemical impedance spectra (EIS) for $BiVO_4$-based photoanodes under (a), (b) without and (c) with Na_2SO_3 as hole scavenger. The solid line was fitted by the ZSimp Win software using the proposed equivalent circuit model.

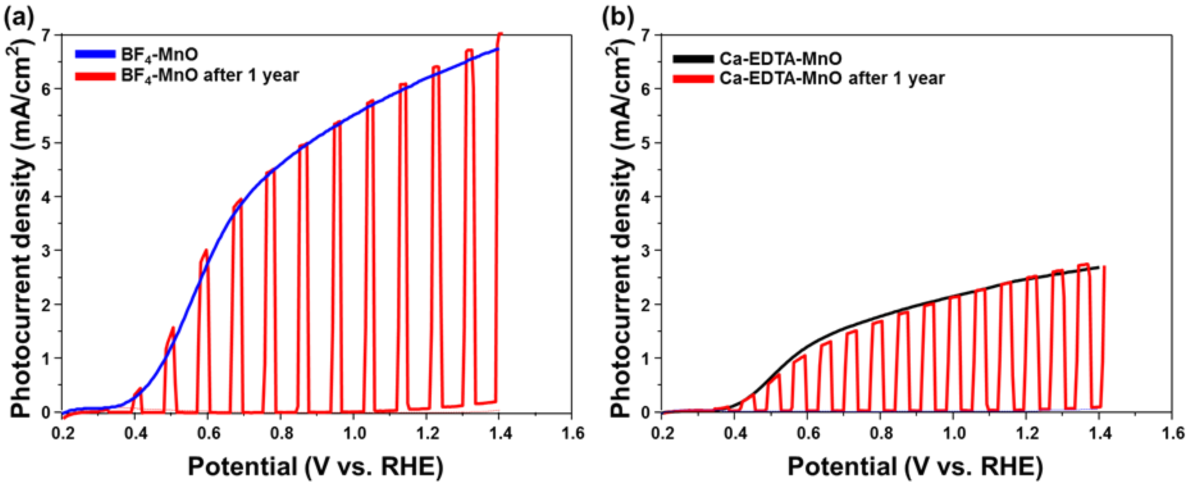

Figure S8. Photocurrent density (a) BF_4-treated and (b) Ca-EDTA-treated MnO NPs loaded on $BiVO_4/WO_3$ anodes synthesized by a year ago, respectively.

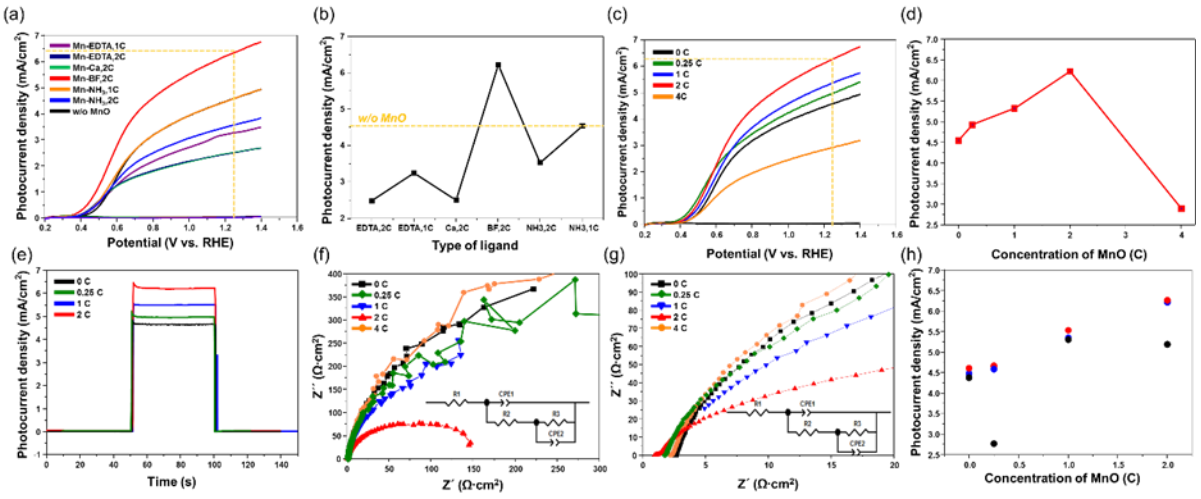

Figure S9. (a) Linear sweep voltammograms of MnO/$BiVO_4$/WO_3 with different ligands and concentration of MnO NPs. (b) Photocurrent density of MnO/$BiVO_4$/WO_3 according to the types of ligand at 1.23 V vs. RHE. (c) Linear sweep voltammograms of BF_4-treated MnO/$BiVO_4$/WO_3 with different concentration of MnO NPs. (d) Photocurrent density of BF_4-treated MnO/$BiVO_4$/WO_3 according to the concentration of ligand at 1.23 V vs. RHE. (e) Amperometric current density-time profiles for MnO/$BiVO_4$/WO_3 with different concentration of MnO NPs at 1.23 V vs. RHE. (f) Electrochemical impedance spectra (EIS) for various concentration of BF_4-treated MnO NPs loaded on $BiVO_4$/WO_3. The solid line was fitted by the ZSimpWin software using the proposed equivalent circuit model. (g) The enlarged EIS plot of various concentration of BF_4-treated MnO NPs loaded on $BiVO_4$/WO_3. (h) Reproducibility of BF_4-treated MnO/$BiVO_4$/WO_3 with different concentration of ligands.

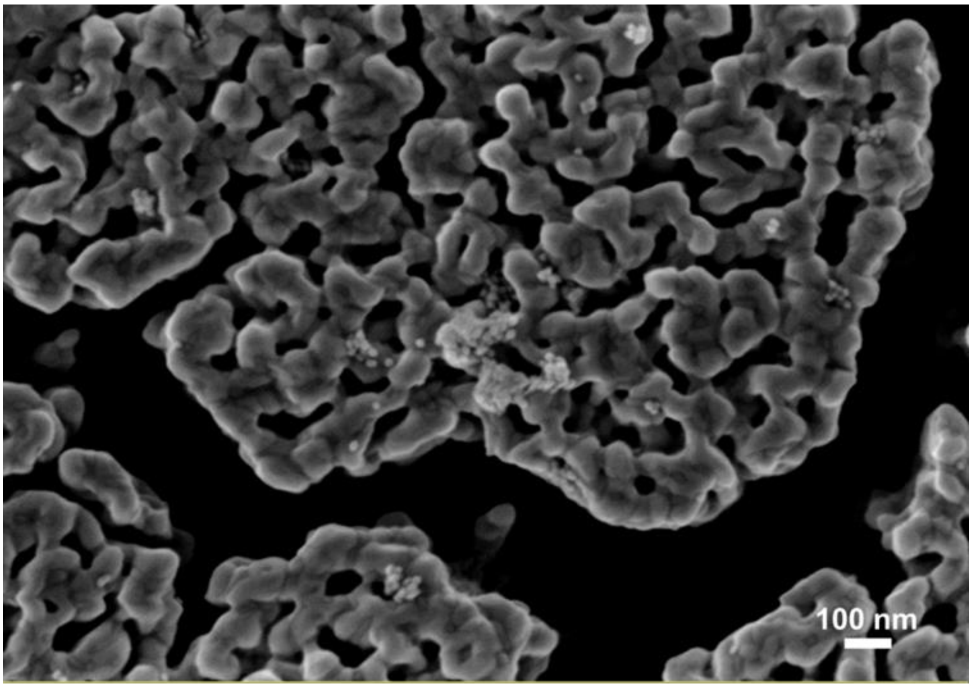

Figure S10. SEM image of $MnO/BiVO_4/WO_3$ with excessive MnO NPs.

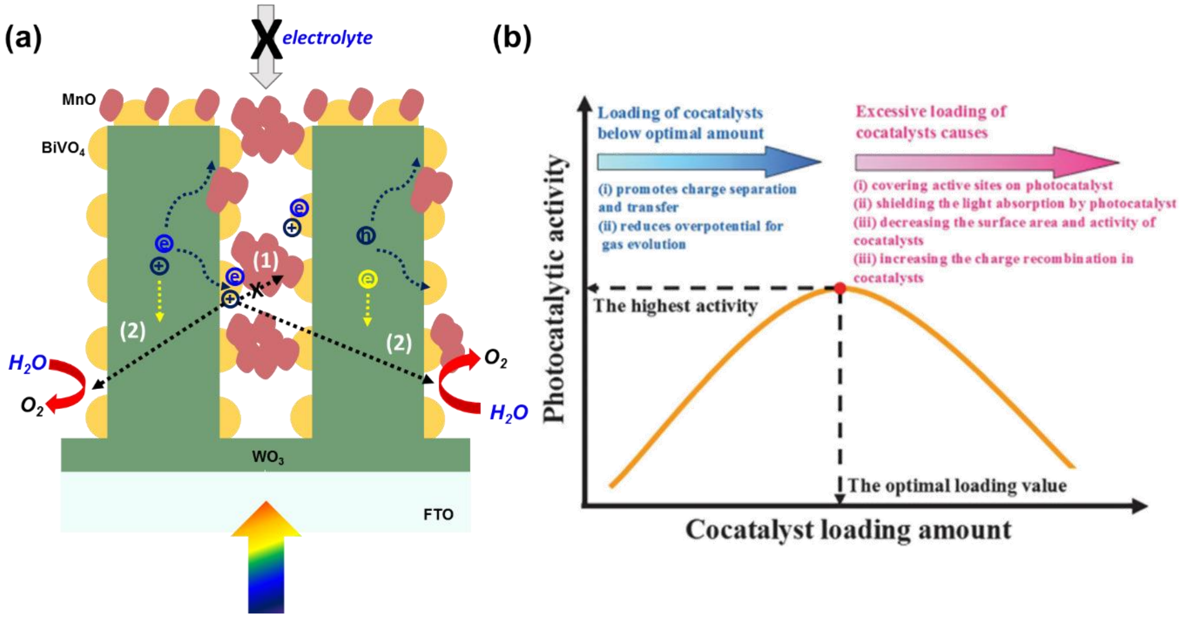

Figure S11. (a) Schematic of charge transfer with excessive co-catalyst, (b) A volcano-type relationship between the loading amount of co-catalyst and the photocatalytic activity of the co-catalyst-loaded semiconductor photoelectrode.[74]

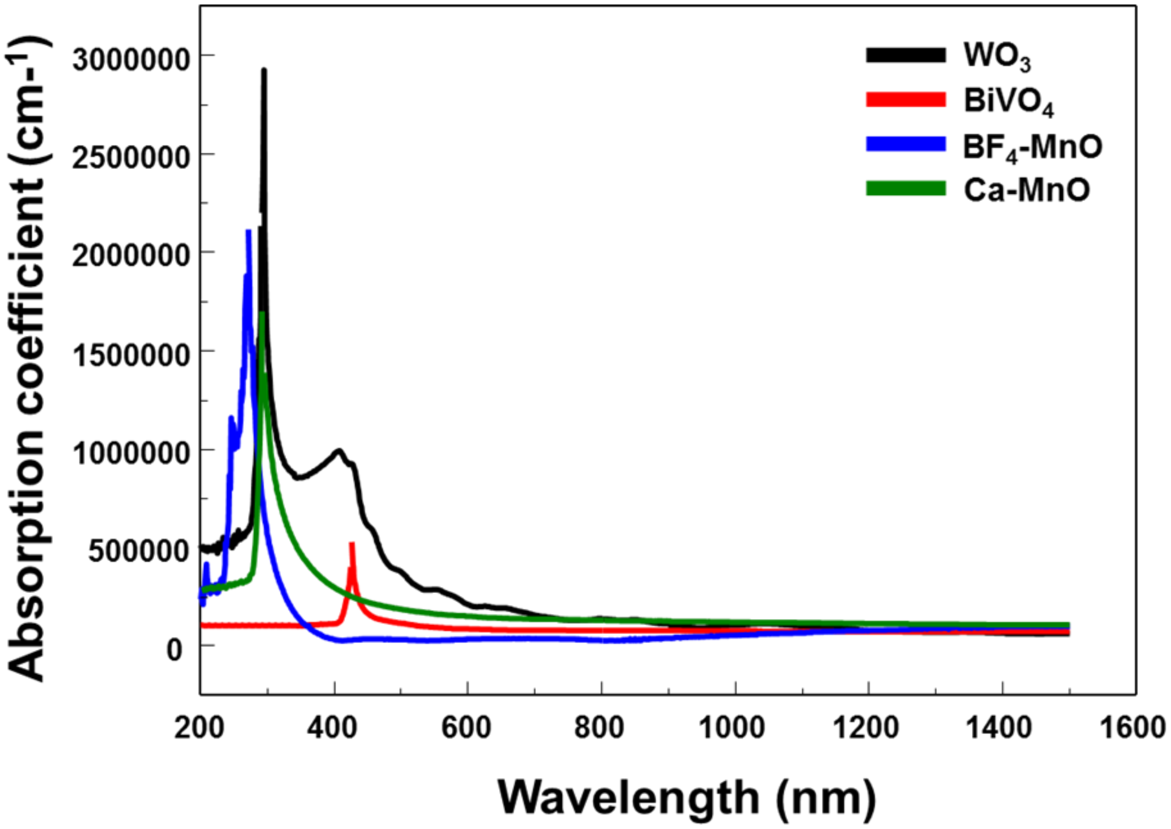

Figure S12. Absorption coefficient (α) calculation of WO_3, $BiVO_4$, BF_4-MnO and Ca-MnO.

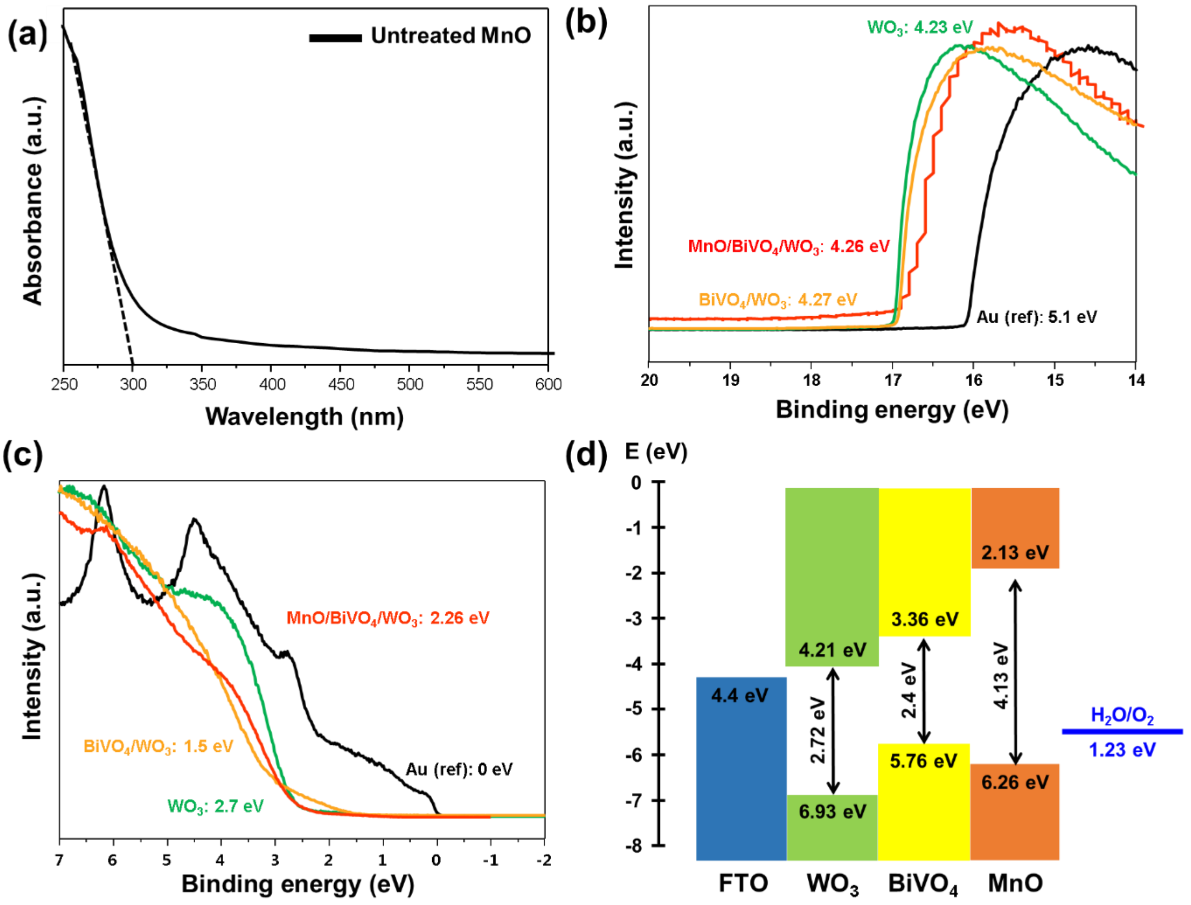

Figure S13. (a) UV-Vis Spectra of untreated $MnO/BiVO_4/WO_3$ (b) The secondary electron emission spectra of the WO_3, $BiVO_4/WO_3$, untreated $MnO/BiVO_4/WO_3$ and reference Au foil electrodes. (c) Valence band spectra, the energy difference between the Fermi level and the valence band maximum (E_F - E_V) of WO_3, $BiVO_4/WO_3$ and untreated $MnO/BiVO_4/WO_3$. (d) Flat band structure of untreated $MnO/BiVO_4/WO_3$.

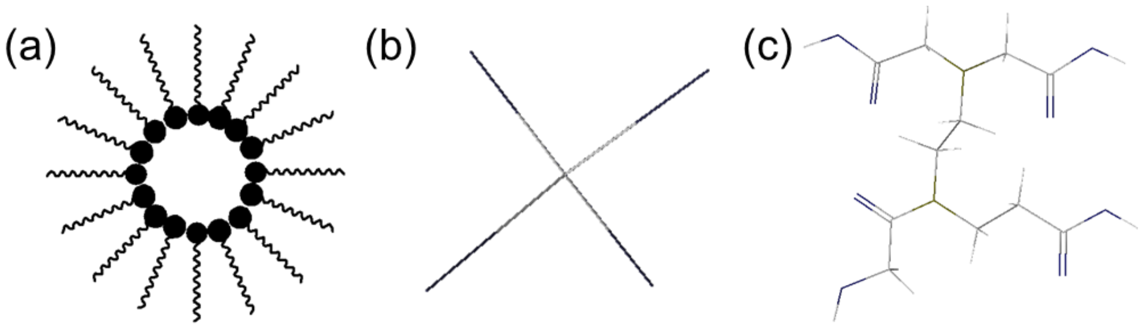

Figure S14. (a) Structure of ligands, (b), (c) Constitutional formula of BF_4 and Ca-EDTA ligands.

Table S1. Comparison of OER activity for various water oxidation catalysts.

Catalyst	Method	Electrolyte	Tafel slope (mV/dec)	Overpotential	Ref
MnO_X	Corrosion method	pH 7	120	N/A	37
MnO_X	Electrodeposition	pH 7	76	0.5 mA/cm^2 @ 565 mV	31
Mn_2O_3 NP	Drop casting	pH 7	120	0.08 mA/cm^2 @ 680 mV	32
Mn_5O_8	Drop casting	pH 7.8	78.7	5 mA/cm^2 @ 580 mV	38
MnO_X	Electrodeposition	pH 13	N/A	N/A	39
MnO_X	Electrodeposition	pH 7	74	N/A	40
MnO_X	Sputtering	pH 13	N/A	0.09 mA/cm^2 @ 300 mV	41
γ-MnOOH	Electrodeposition	pH 0.3	N/A	N/A	43
α-Mn_2O_3	Electrodeposition	pH 13	N/A	1 mA/cm^2 @ 300 mV	42
MnO	Atomic layer deposition	pH 13	N/A	0.4 mA/cm^2 @ 300 mV	43
MnO_X	Spin coating	pH 13	49	0.002 mA/cm^2 @	45

				300 mV	
β-MnO_2	Screen-printed	pH 13	N/A	10 mA/cm^2 @ 300 mV	46
MnO NCs	Spin coating	pH 7	N/A	5 mA/cm^2 @ 530 mV	32
Co-Pi	Electrodeposition	pH 7	N/A	5 mA/cm^2 @ 570 mV	47
MnO_X	Electrodeposition	pH 7	N/A	1 mA/cm^2 @ 600 mV	48
Mn_3O_4	Spin coating	pH 7	N/A	40 μA/cm^2 @ 650 mV	32
Mn_2O_3	Spin coating	pH 7	N/A	40 μA/cm^2 @ 490 mV	32
MnO_2	Spin coating	pH 7	N/A	40 μA/cm^2 @ 630 mV	32
IrO_X	Electrodeposition	pH 7	N/A	5 mA/cm^2 @ 310 mV	49

Table S2. Recent reports on the $BiVO_4$-based photoanodes for solar water oxidation (2011-2018).

Year	Photoelectrode	Electrolyte	Performance	method	ref
2018	FeOOH/NiOOH/$BiVO_4$	0.2 M Na_2SO_3 (pH 9.5)	6.22 mA/cm^2 (1.23 V_{RHE})	ED	77
2018	cobalt phosphate /ethced $BiVO_4$	0.5 M KPi + H_2O_2	6.1mA/cm^2 (1.23 V_{RHE})	laser ablation + PED	19
2017	NiOOH/FeOOH/CQD/$BiVO_4$	0.5 M KPi + 0.5 M Na_2SO_3	6.54 mA/cm^2 (1.23 V_{RHE})	ED + PED	77
2017	CoBi/$BiVO_4$	1 M BK_3O_3 (pH 9.5)	3.2 mA/cm^2 (1.23 V_{RHE})	Hydrothermal + PED	20
2017	$BiVO_4$/WO_3/SnO_2	0.5 M KPi + H_2O_2	3.11 mA/cm^2 (1.23 V_{RHE})	sol−gel + spin-coating	59
2017	cobalt phosphate /$BiVO_4$/WO_3	0.5 M Na_2SO_4 (pH 6.8)	1.5 mA/cm^2 (1.23 V_{RHE})	Sol gel + ED + PED	60
2016	$BiVO_4$/WO_3	0.5 M KPi + 0.5 M Na_2SO_3	4.0 mA/cm^2 (1.23 V_{RHE})	polymer assisted decomposition deposition + MOD	61
2016	$BiVO_4$/WO_3	0.5 M KPi + 1 M Na_2SO_3 (pH 7.2)	4.55 mA/cm^2 (1.23 V_{RHE}) ~ 80 % IPCE at 460 nm	GLAD + Pulsed ED	4
2015	FeOOH/NiOOH/ N_2-treated $BiVO_4$	0.5 M KPi + 1 M Na_2SO_3 (pH 7.2)	4.16 mA/cm^2 (0.6 V_{RHE}) Significant absorption (wavelength > 550nm)	ED + PED	62
2015	Co-Ci/H, 3% Mo:$BiVO_4$	0.1 M KCi (CO_2 purged) (pH 7)	4.8 mA/cm^2 (1.23 V_{RHE}) ~ 80 % IPCE at 420 nm (1.23 V_{RHE})	MOD + PED	66
.2014	Co-Pi/$BiVO_4$/WO_3-NRs	0.5 M Na_2SO_4 (pH 7)	3.2 mA/cm^2 (1.23 V_{RHE}) ~ 60 % IPCE at 400 nm	Multi-magnetron GLAD + sputtering	16
2014	NiOOH/FeOOH/$BiVO_4$	0.5 M KPi + 1 M Na_2SO_3 (pH 7.2)	4.2 mA/cm^2 (1.23 V_{RHE}) ~ 60 % IPCE at 420 nm	ED + PED	21
2014	$CaFe_2O_4$/$BiVO_4$	0.5 M Na_2SO_4 (pH 6.5)	~ 1.0 mA/cm^2 (1.23 V_{RHE}) 20−22%, IPCE at 340−450 nm	MOD + electrophoretic deposition	67
2014	W-$BiVO_4$/WO_3-NWs	0.5 M KPi	3.1 mA/cm^2 (1.23 V_{RHE}) ~60% IPCE at 300–450 nm	Flame vapor deposition + drop-casting	17
2014	NiOOH/FeOOH/W, Mo:$BiVO_4$/helix WO_3	0.5 M K_2SO_4 (pH 7)	5.35 mA/cm^2 (1.23 V_{RHE}) 90 % IPEC	GLAD + dropping + PED	65
2014	Co-Pi/$BiVO_4$/ZnO	0.2 M Na_2SO_4 (pH 6.5)	2.5 mA/cm^2 (1.23 V_{RHE})	Spray pyrolysis	68

			~ 47 % IPCE at 410 nm<br>0.88 % photo-conversion efficiency		
2014	Co-P2012015i/BiVO$_4$/WO$_3$	0.1 M KPi<br>+<br>0.5 M Na_2SO0_3<br>(pH 7)	3.3 mA/cm^2 (1.23 V_{RHE})<br>50~65 % IPCE at 300-470 nm	Hydrothermal<br>+<br>Spin coating<br>+<br>ED	13
2013	Co-Pi/Gradient W-BiVO$_4$	1 M KPi<br>(pH 7 .3)	~ 3 mA/cm^2 (1.23 V_{RHE})<br>0.6 η_{sep} (1.23 V_{RHE})	Spray pyrolysis	69
2012	BiVO$_4$/SnO$_2$/WO$_3$	0.1 M Na_2SO_4	~ 2.7 mA/cm^2 (1.23 V_{RHE})<br>43 % IPCE at 420 nm	Wet coating	64
2011	BIVO$_4$/WO$_3$	0.5 M Na_2SO_4<br>(Ph 6.6)	1.74 mA/cm^2(1.23 V_{RHE})<br>40 % IPCE at 420 nm	Polymer assisted direct deposition + calcination	14
**Our work**	BF$_4$-treated MnO/BiVO$_4$/WO$_3$	0.5 M KPi<br>+<br>1 M Na_2SO_3<br>(pH 7.2)	6. 25 mA/cm^2 (1.23 V_{RHE})<br>~ 80 % IPCE at 460 nm	GLAD<br>+<br>Pulsed ED<br>+<br>Spin coating	

Table S3. Circuit analysis of the EIS spectra for $BiVO_4$-based photoelectrodes with different ligands (a) under water oxidation and (b) and (c) sulfite oxidation.

(a)

**($R/\Omega\cdot cm^2$)**	**w/o MnO**	**BF_4-MnO**	**Ca-MnO**
R_s **(series resistance)**	6.77	1.71	2.27
R_{ct1} **(semiconductor interfaces)**	72.62	6.33	1335.34
R_{ct2} **(semiconductor/ electrolyte interface)**	572.67	192.12	291.2

(b)

**($R/\Omega\cdot cm^2$)**	**$BiVO_4$**	**w/o MnO**	**BF_4-MnO**	**Ca-MnO**
R_s **(series resistance)**	1.68	2.59	1.04	1.95
R_{ct1} **(semiconductor interfaces)**	196.2	22.36	3.09	953.80
R_{ct2} **(semiconductor/ electrolyte interface)**	768.85	898.11	129.06	134.90

(c)

**CPE ($/10^{-6}$ F)**	**$BiVO_4/WO_3$**	**BF_4-MnO/$BiVO_4/WO_3$**	**Ca-EDTA-MnO/$BiVO_4/WO_3$**
**CPE1 (semiconductor interfaces)**	0.71	4.67	3.21
**CPE2 (semiconductor/ electrolyte interface)**	8.67	15.75	6.13

Table S4. Circuit analysis of the EIS spectra for $MnO/BiVO_4/WO_3$ photoelectrodes with different concentration of MnO.

**($R/\Omega\cdot cm^2$)**	**0 C**	**0.25 C**	**1 C**	**2 C**	**4 C**
R_s **(series resistance)**	2.59	1.78	1.73	1.04	2.293
R_{ct1} **(semiconductor interface)**	22.36	13.38	5.24	3.09	852.11
R_{ct2} **(semiconductor/electrolyte interface)**	898.11	717.95	583.41	129.06	172.19

Table S5. (a) Energy level of valence band and work function of FTO, WO_3, $BiVO_4/WO_3$, BF_4-MnO/$BiVO_4/WO_3$ and Ca-MnO/$BiVO_4/WO_3$ investigated by ultraviolet photoemission spectroscopy (b) Optical band gap of WO_3, $BiVO_4$, BF_4 treated MnO and Ca treated MnO measured by UV-visible spectroscopy.

(a)

Materials	E_F - E_V	Work function
**FTO**	N/A	4.4 eV
**WO_3**	2.7 eV	4.23 eV
**$BiVO_4/WO_3$**	1.5 eV	4.27 eV
**BF_4-MnO/$BiVO_4/WO_3$**	1.5 eV	4.2 eV
**Ca-EDTA- MnO/$BiVO_4/WO_3$**	1.8 eV	4.2 eV
**Untreated MnO/$BiVO_4/WO_3$**	2.26 eV	4.26 eV

(b)

Materials	Optical band gap (ref)	Optical Band gap (measure)
**FTO**	N/A	N/A
**WO_3**	2.8 eV	2.72 eV
**$BiVO_4$**	2.4 eV	2.4 eV
**BF_4-MnO**	4.2 eV	4.3 eV
**Ca-EDTA-MnO**	4.2 eV	4.0 eV
**Untreated MnO/$BiVO_4/WO_3$**	4.0 eV	4.13 eV
